# RNA polymerase II pausing can be retained or acquired during activation of genes involved in the epithelial to mesenchymal transition

**DOI:** 10.1093/nar/gkv263

**Published:** 2015-03-27

**Authors:** Ann Samarakkody, Ata Abbas, Adam Scheidegger, Jessica Warns, Oscar Nnoli, Bradley Jokinen, Kris Zarns, Brooke Kubat, Archana Dhasarathy, Sergei Nechaev

**Affiliations:** 1 Department of Basic Sciences, University of North Dakota School of Medicine, Grand Forks, ND 58202, USA; 2 Department of Computer Sciences, University of North Dakota, Grand Forks, ND 58202, USA

## Abstract

Promoter-proximal RNA polymerase II (Pol II) pausing is implicated in the regulation of gene transcription. However, the mechanisms of pausing including its dynamics during transcriptional responses remain to be fully understood. We performed global analysis of short capped RNAs and Pol II Chromatin Immunoprecipitation sequencing in MCF-7 breast cancer cells to map Pol II pausing across the genome, and used permanganate footprinting to specifically follow pausing during transcriptional activation of several genes involved in the epithelial to mesenchymal transition (EMT). We find that the gene for EMT master regulator Snail (*SNAI1*), but not Slug (*SNAI2*), shows evidence of Pol II pausing before activation. Transcriptional activation of the paused *SNAI1* gene is accompanied by a further increase in Pol II pausing signal, whereas activation of non-paused *SNAI2* gene results in the acquisition of a typical pausing signature. The increase in pausing signal reflects increased transcription initiation without changes in Pol II pausing. Activation of the heat shock *HSP70* gene involves pausing release that speeds up Pol II turnover, but does not change pausing location. We suggest that Pol II pausing is retained during transcriptional activation and can further undergo regulated release in a signal-specific manner.

## INTRODUCTION

The process of epithelial to mesenchymal transition (EMT) involves the conversion of cells from an attached, epithelial morphology to a migratory, stem cell-like phenotype. EMT is essential in normal development, but when activated in cancer cells it can lead to metastasis (1,2). EMT is a complex process involving several genes, many of which are potential targets for anti-cancer therapy, including *SNAI1, SNAI2, ZEB1* and *HSP70* (3–5). The products of *SNAI1* (Snail) and *SNAI2* (Slug) genes are master regulators of EMT and their elevated levels confer poor prognosis in breast cancer (6,7). The Snail and Slug proteins promote EMT through repressing genes such as E-cadherin (*CDH1*), (which encodes a cell–cell adhesion protein) by binding to their promoter regions and recruiting repressor complexes including histone deacetylases (HDACs) (8–10). One of the challenges in the prevention and treatment of metastatic cancers is that commonly used drugs, especially broad-spectrum agents including HDAC inhibitors, can act through multiple mechanisms [reviewed in (11)] and have variable effects on different cell types (12,13).

Promoter-proximal Pol II pausing occurs when the enzyme initiates transcription of a gene, but stops before completing the first ∼100 nucleotides of its RNA. Pausing has been shown to be prevalent in metazoans, particularly among genes involved in stimulus responses, cell differentiation, development and formation of memory [reviewed in (14)]. Despite its connections to various regulatory processes, however, the contribution of Pol II pausing to gene regulation remains to be understood. Recent studies rule out the most simplistic explanations by demonstrating that pausing does not repress genes (15,16) and does not enable more rapid gene activation (17). Instead, the outcomes of pausing appear to be more complex, including fine-tuning basal activity of genes (18), facilitating synchronous responses to developmental signals (19–21) or faster recovery after activation (22). Mechanistically, paused Pol II has been shown to counteract promoter-proximal nucleosomes (23–25), pointing to a connection between pausing and epigenetic regulation.

One unresolved question important for understanding of transcriptional responses to stimuli is the fate of Pol II pausing during gene activation. A potential discrepancy arising from recent work is that, on the one hand, Pol II pausing is recognized to occur on genes prior to activation (26,27), which implies that pausing should be released when the gene is activated. Indeed, analysis of *Drosophila* heat shock genes demonstrated that the half-life of paused Pol II in the basal state, ∼5 min (28–30), is reduced to several seconds at the conditions of heat shock activation, such that pause release contributes to their robust upregulation (28,29). On the other hand, paused Pol II is also known to be associated with active genes (15,31). Furthermore, recent chromatin immunoprecipitation (ChIP) and global run-on analyses indicate that Pol II signal proximal to the 5′-ends of genes can increase with gene activation (17,32–33), suggesting that Pol II can remain near promoters. However, because these methods detect promoter-proximal, but not necessarily paused polymerase, the fate of pausing, which includes its dynamics and location during gene activation, remains uncertain.

Here, we characterized Pol II pausing in human MCF-7 breast cancer cells. As genes have been proposed to activate through two distinct mechanisms including Pol II recruitment or pause release (33), we followed Pol II pausing during activation through both mechanisms. To do that, we performed short capped RNA (scRNA) and paired-end ChIP sequencing to map promoter-proximally paused Pol II globally, and specifically followed pausing on several genes involved in EMT using permanganate footprinting. Before activation, Pol II pausing location is similar on genes across the genome, peaking at ∼33–35 nt downstream of the transcription start site (TSS). Using the gene for the transcription factor Snail as a model system, we show that its activation in response to stimuli including beta-estrogen, transforming growth factor-beta1 (TGF-beta) and an HDAC inhibitor trichostatin A (TSA) is accompanied by retention of pausing at its original location without changes in pausing duration. We also show that activation of a human *HSP70* gene by heat shock through pause release changes the duration, but not the location of paused Pol II. The findings indicate that pausing can be retained during gene activation. We suggest that stable pausing during gene activation provides a regulatory platform to orchestrate transcriptional responses of cells to stimuli.

## MATERIALS AND METHODS

### Cell culture

All cell lines used here were obtained from the American Type Culture Collection and checked for mycoplasma contamination using MycoAlert kit (Lonza) before use. Cells were cultured in DMEM/F-12 medium (Life Technologies) supplemented with 10% fetal bovine serum (FBS) (Gibco) at 37°C in 5% CO_2_ atmosphere. Treatments were done using 1-μM TSA and/or 2.5-μM triptolide (TRP) (Sigma). Cells for 17β-estradiol (E2) treatments were maintained in phenol red free DMEM/F-12 media supplemented with charcoal–dextran-stripped 10% FBS (Atlanta Biologicals) and ITS (insulin-transferrin-selenium, Lonza), and were treated with ethanol (vehicle) or 1-nM E2 (Sigma). NMuMG cells were treated with 5-ng/ml recombinant TGF-beta (Sigma). Heat shock was done by rapidly placing a culture dish into a water bath preheated to 42°C for 1 min followed by incubation in a dry incubator at 42°C.

### Chromatin immunoprecipitation

ChIP was performed as previously described (34) with modifications. Approximately 5 × 10^7^ cells were crosslinked in 1% formaldehyde in serum free DMEM/F-12 media for 5 min at room temperature followed by quenching with glycine. Cells were washed with phosphate buffered saline and lysed on ice in 0.5% sodium dodecyl sulphate Radioimmunoprecipitation assay (RIPA) buffer. The lysate was sheared using the Covaris S220 sonicator for 6 min using the high cell program (output = 140, duty cycle = 5). Chromatin was incubated with protein A+G magnetic beads (Life Technologies) in the presence of an appropriate antibody: N-20, Santa Cruz for Pol II or H-311 for HSF1. Precipitated DNA was quantified by real time polymerase chain reaction (PCR) and normalized against a standard curve generated using serial dilutions of the input for each pair of primers. Each experiment was performed using a minimum of three independent biological replicates for quantitative PCR (qPCR) analysis and two independent biological replicates for ChIP-sequencing. For ChIP-sequencing, precipitated DNA (10 ng as determined by Qubit fluorometer) was used to prepare a library using Illumina ChIP-seq kit using 14 cycles of PCR amplification. Libraries were checked for enrichment with gene-specific primers and positive and negative control sets (Active Motif), and quantified for cluster generation using KAPA Library Quantification Kit. Sequencing was done using Illumina miSeq in 75-bp paired end format.

### scRNA preparation

scRNA libraries were prepared as described (35), with modifications. Approximately 5 × 10^6^ cells were used to extract nuclei. Thirty microgram of trizol-extracted nuclear RNA was subjected to size selection on a 15% polyacrylamide Tris-borate-EDTA (TBE) gel containing 7-M urea, extracted using crush and soak method and ethanol precipitated. Capped RNAs were isolated by sequential treatment of size-selected RNA with 5′-polyphosphatase, 5′-terminator exonuclease and tobacco acid pyrophosphatase (TAP) (Epicentre). Phenol–chloroform and chloroform extraction followed by ethanol precipitation was done between each enzyme treatment. RNA was used to prepare a library using Illumina Tru-seq Small RNA kit using 18 cycles of amplification. Libraries were validated by cloning into pBlueScript vector and Sanger-sequencing a minimum of 10 clones to verify the inserts and quantified using KAPA Library Quantification Kit. Sequencing was done on a MiSeq using ‘Generate fastq files’ RNA LT routine in 50-bp paired end format.

### Data analysis

Matching fastq file pairs were aligned against hg19 reference genome using ‘bowtie’ (36). Top 30% of mRNA genes based on Pol II ChIP signal enrichment in the promoter regions were taken for further analysis (see the Supplementary methods). ScRNA TSSs were defined as the position with the highest number of scRNA 5′-end hits within −500 to +500-nt interval around the annotated mRNA TSS. Enrichment of sequence motifs was visualized using Weblogo (37). Data on individual genes were visualized using UCSC genome browser (38). The data are accessible through NCBI's Gene Expression Omnibus (39) under accession number GSE67041.

### Reverse transcription qPCR

Total RNA was extracted from cells using RNeasy kit (Qiagen) and checked for integrity using agarose gel electrophoresis. One microgram of RNA was used to synthesize cDNA using random hexamer priming and SSRT III reverse transcriptase (Life Technologies) followed by qPCR using QuantiTect primer assays (Qiagen). Data were normalized against *GAPDH* and *ACTB* gene transcripts. Data were derived from at least three independent biological replicates and are shown as mean ± SEM values.

### Permanganate footprinting

Permanganate treatment of cells and subsequent preparation of genomic DNA was done as described previously for *Drosophila* (35,40). One hundred fifty nanogram of piperidine-treated DNA was used in ligation-mediated PCR (Supplementary Figure S1). The initial primer extension with gene-specific primer A was done in 20 μl of Phusion buffer HF (2 min 98°C, 5 min 50°C and 3 min 72°C) using Phusion DNA polymerase, followed by ligation of double-stranded linker A′:B at 16°C overnight. After ethanol precipitation, DNA was amplified in 100 μl of 1xPhusion HF buffer using gene-specific primer B and universal Linker A′ for 22 cycles of PCR (98°C 15 s, 54°C 30 s, 72°C 30 s). ^32^P 5′-end-labelled primer (primer C) was then added and two more cycles of amplification with annealing temperature of 59°C were performed. Reactions were phenol/chloroform extracted, ethanol precipitated, resuspended in loading dye (8-M urea, 1xTBE, bromophenol blue dye) and resolved on a denaturing 7% urea-TBE gel followed by radioautography. The number of PCR cycles was adjusted to be within the linear range of the method and was confirmed using titrations of cycle numbers (Supplementary Figure S1).

### Synthetic bubble DNA template construction

The 186-bp upstream and 146-bp downstream double-stranded DNA fragments were prepared by annealing synthetic Ultramer oligonucleotides (IDTDNA) containing appropriate 5′-phosphate modifications and purifying on a 2% agarose gel. The bubble was created by substituting the sequence in the bottom strand oligonucleotide corresponding to *SNAI1* positions +32 and +51 from the annotated TSS. The double-stranded or bubble upstream fragment was ligated to the common downstream fragment using T4 DNA ligase to generate a template spanning positions −33 to +298 of *SNAI1* gene. After additional gel purification, the fully double-stranded and bubble templates were combined to generate a series of templates with a defined proportion of the bubble, which were then permanganate footprinted as above. Ratios of templates were verified prior to combining using both the Nanodrop and by qPCR using primers flanking the ligation site.

### Small RNA northern blotting

Small RNA northern blotting was done as previously described (41) with modifications. One hundred-fifty microgram of trizol-extracted total RNA was subjected to small RNA selection using miRNA QIaprep kit (Qiagen) followed by treatment with TAP when indicated. Small RNA was then loaded on a 15% urea-TBE gel alongside gamma^32^P-labeled Decade marker (Life Technologies) and transferred onto a neutral nylon membrane (Hybond-NX, GE Healthcare). 5′-Phosphate selective crosslinking of RNA to the membrane was done using 1-Ethyl-3-[3-dimethylaminopropyl]carbodiimide hydrochloride as described (42). Membranes were probed with StarFire probes (IDTDNA) labeled with α^32^PdATP per manufacturer's instructions.

## RESULTS

### Promoter-proximal pausing in MCF-7 cells across the genome

**Figure 1.**
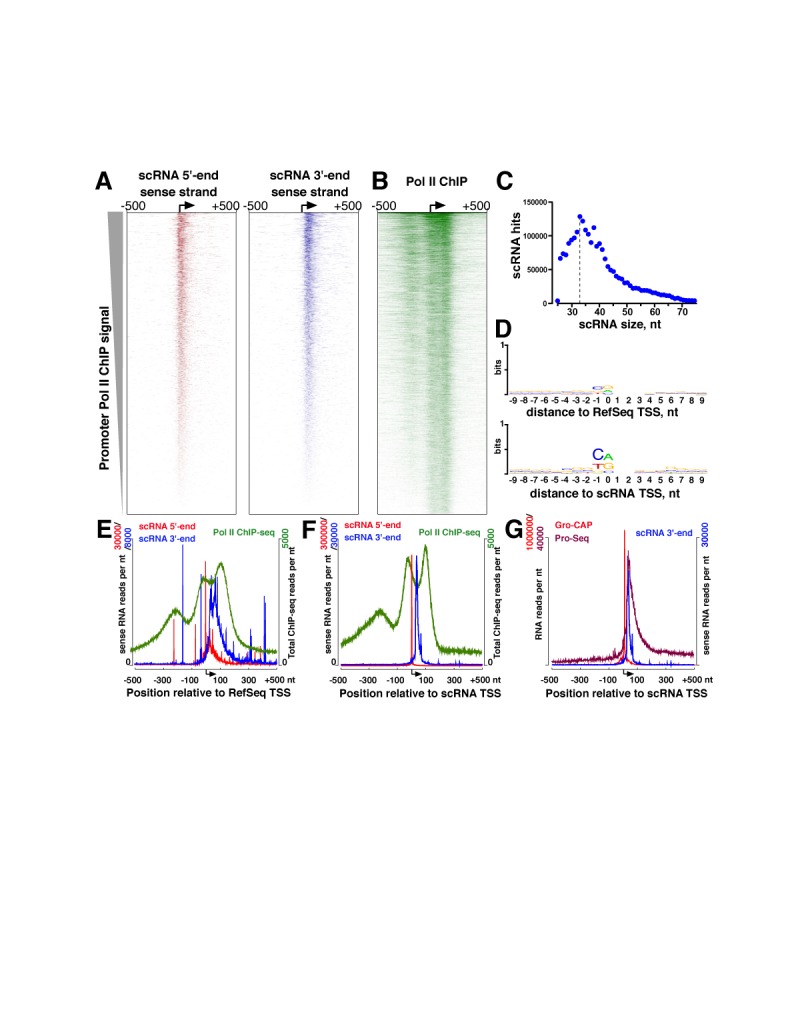
Pol II pausing in MCF-7 cells. (**A**) Heatmaps showing scRNAs for 7302 genes with the highest promoter Pol II enrichment, in the region of ±500 nt around their RefSeq (annotated) TSSs. The position of the TSS is shown by an arrow. Only the sense strand scRNAs are shown. (**B**) Heatmap showing Pol II ChIP-sequencing profiles for the same genes sorted as in (A). (**C**) A metagene plot showing the frequency distribution of lengths of scRNAs based on distances between R1 and R2 paired-end sequencing read positions. (**D**) Weblogo (37) enrichment of sequence motifs around RefSeq TSSs (top) and scRNA-derived TSSs (bottom). (**E**) A metagene profile of Pol II ChIP sequencing and scRNA signal centered around RefSeq TSSs. (**F**) Metagene profiles are the same as in (E), re-centered around the scRNA TSSs. (**G**) Metagene profiles for the same genes derived from Pro-Seq and Gro-CAP reads in K562 cells (available under accession codes in Gene Expression Omnibus Accession numbers GSM1480321 and GSM1480327, respectively).

We mapped promoter-proximal Pol II pausing in MCF-7 cells by sequencing scRNAs extracted from MCF-7 cell nuclei. Global scRNA sequencing was performed in paired end format to determine both 5′- and 3′-ends of the same RNAs. The 5′-ends of these scRNAs align well to the annotated TSSs of mRNA genes (Figure 1A and E). The 3′-ends of scRNA, which is indicative of paused Pol II positions (35), have the predominant length of 33–35 nt downstream of the annotated TSS (Figure 1A and C). We also noted the recently described phenomenon of divergent transcription, with a broad distribution of antisense scRNAs centered ∼150 nt upstream of the gene TSSs (Supplementary Figure S2), indicating that divergent transcription involves Pol II pausing (43,44).

Recent high-resolution re-analysis of existing Pol II ChIP-sequencing data stipulated the existence of two polymerase peaks centered at the TSS and 110 nt downstream of the TSS (45), in an apparent contradiction to Pol II positioning suggested by our scRNA sequencing in MCF-7 cells as well as previous work in other systems (15,26,35,46). Because single-end ChIP-sequencing carries an uncertainty in centering of the read positions and thus hampers resolution of closely located peaks, we performed Pol II ChIP-sequencing in paired end format to determine read center positions directly. We did find evidence of two peaks of Pol II centered at around TSS and +110 nt from the TSS, which are distinct from the upstream peak arising from divergent transcription (Figure 1B and E). To determine whether the peaks near the TSS may have arisen from misannotation of TSSs, we re-centered the heatmaps based on the location with the highest number of scRNA 5′-ends, which were previously shown to define TSSs in *Drosophila* (35), and also match recently defined TSSs in human cells in a different cell line (44) (Figure 1G). Re-centering of the TSSs based on scRNAs revealed sharpening of metagene profiles of scRNAs (Figure 1F and Supplementary Figure S3), as expected, and also increased the enrichment of the initiator-like motif (47) around the scRNA TSSs (Figure 1D and Supplementary Figure S4), indicating that scRNAs define the start sites for the majority of mRNA gene transcription events. At the same time, scRNA TSSs further emphasized, not diminished, the two ChIP-sequencing peaks (Figure 1F and Supplementary Figure S3). Indeed, the 3′-end positions of scRNAs do not correspond to either of the ChIP-seq peaks, and are located between them (Figure 1F and Supplementary Figure S5). Thus, the positioning of promoter-proximally paused Pol II in MCF-7 cells is consistent with that observed in *Drosophila* (35,48), suggesting that the mechanisms of pausing are conserved across metazoans.

### Promoter-proximal pausing on EMT-related genes in MCF-7 cells before activation

**Figure 2.**
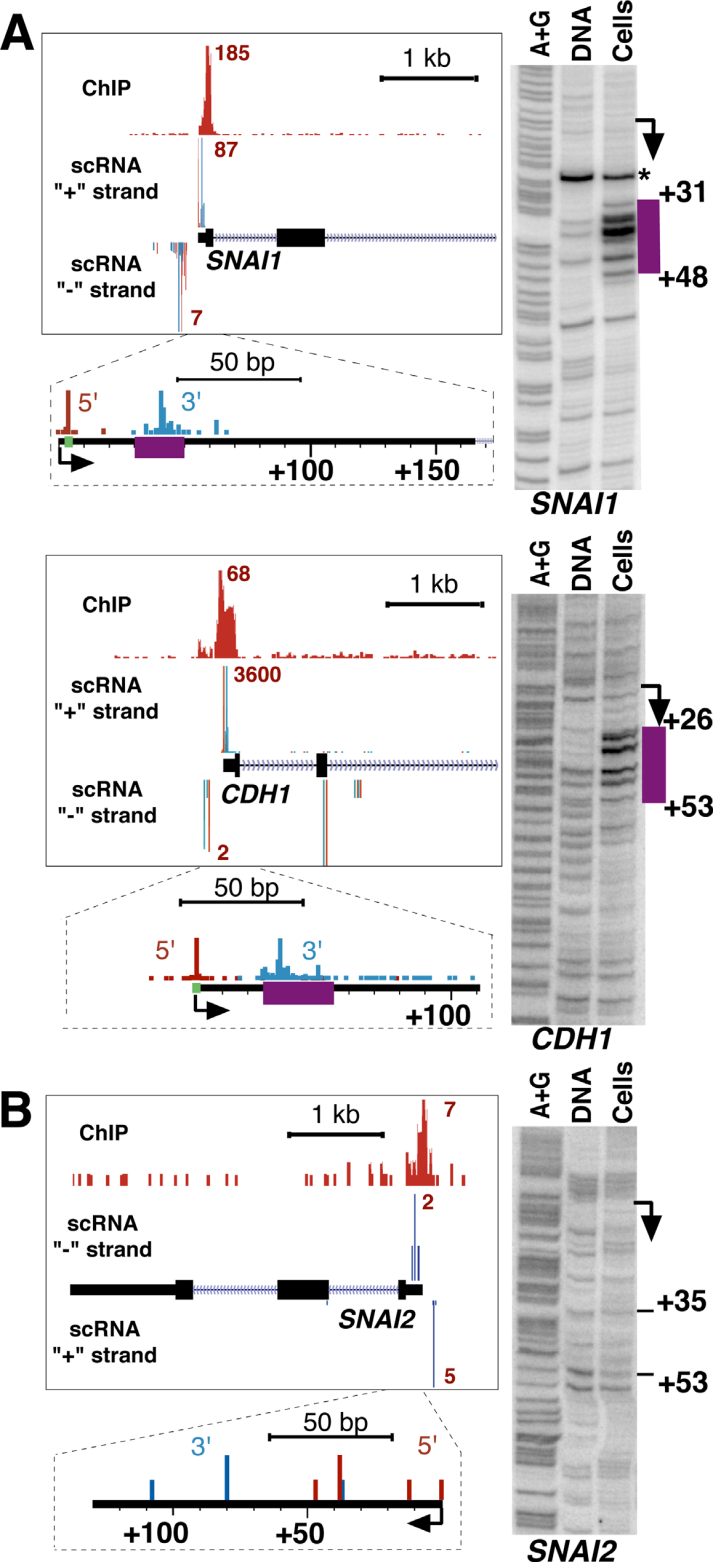
Pol II pausing on individual genes. (**A**) *SNAI1* and *CDH1* genes show evidence of Pol II pausing. (**B**) *SNAI2* gene does not show evidence of pausing. For each gene, a UCSC genome browser (38) view is shown, with positions of Pol II ChIP-sequencing bins and the positions of 5′ and 3′-RNAs indicated. For scRNAs, plus and minus strand matches are shown as separate tracks. Numbers near each peak represent the maximum number of sequence reads in a bin (25-bp bin for ChIP-sequencing, 1-bp bin for scRNA) within the displayed interval. An inset below each gene shows a zoomed-in view of scRNA tracks around the TSS. Permanganate footprinting of the same gene is shown on the right, alongside naked DNA and A/G ladder. Annotated TSSs are shown with arrows and TSS start sites are shown in green. The region of permanganate reactivity is shown alongside the gel and on the gene scheme in purple. An asterisk at the *SNAI1* gene indicates a non-specific band present both in cells and naked DNA.

Based on our scRNA and Pol II ChIP-sequencing, we noted that of the two master regulators of EMT, *SNAI1* shows high Pol II enrichment at the promoter whereas *SNAI2* does not. To detect pausing on these genes directly and distinguish it from other transcriptional intermediates, we used permanganate footprinting. The approach relies on the ability of permanganate to oxidize unpaired thymine bases on DNA and thus can visualize the location of paused Pol II independent of RNA (40,49). Using permanganate footprinting, which we optimized here for use in mammalian cells, we show that *SNAI1* and *CDH1* genes contain Pol II pausing signatures within their promoter-proximal regions (Figure 2A), whereas *SNAI2* gene shows no detectable pausing (Figure 2B). These data are consistent with the magnitude of Pol II ChIP-qPCR (Supplementary Figure S6, untreated) and ChIP-sequencing signal near each promoter (Figure 2). We also note that the scRNA 3′-ends obtained by global sequencing closely match the locations of the transcription bubble observed by permanganate footprinting on each of the genes tested (Figure 2A), indicating that scRNAs correctly map promoter-proximal pausing. We observed no evidence of a transcription bubble indicative of a stable open promoter complex around the TSSs on these genes, even when permanganate treatment was performed directly in growth media at 37°C to exclude a possibility of transcription bubble collapse that may have occurred as cells are handled on ice (Supplementary Figure S7). We also found no significant permanganate reactivity downstream of the pausing site in the region of 100–150 nt from these TSSs (Supplementary Figure S8). Thus, Pol II pausing takes place in the promoter-proximal regions of *SNAI1*, but not *SNAI2*, gene before activation.

### Activation of *Snail* and *Slug* genes increases Pol II pausing signal

**Figure 3.**
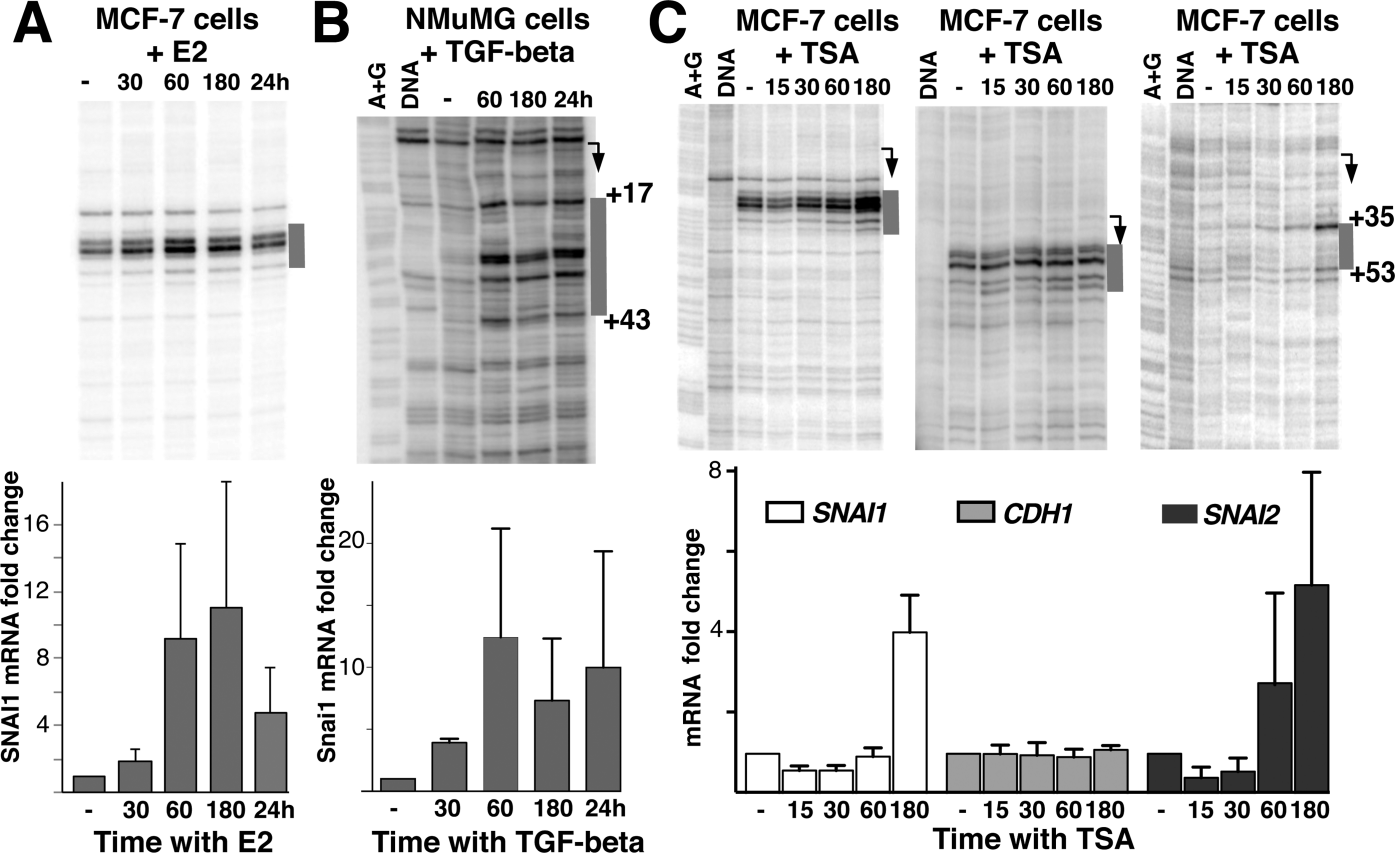
Pol II pausing signal is increased with gene activation. (**A**) MCF-7 cells treated with E2 and probed with permanganate footprinting (top) and RT-qPCR to detect mRNA (bottom). Time of treatment is indicated in minutes unless indicated otherwise. RT-PCR represents an average of four independent replicates. (**B**) Mouse NMuMG cells treated with TGF-beta. RT-PCR and permanganate footprinting represents an average of three independent replicates. (**C**) MCF-7 cells treated with TSA. RT-PCR represents an average of four independent replicates. The regions of permanganate reactivity emerging during activation of mouse *Snai1* and human *SNAI2* genes are shown alongside the gel in gray.

The paused *SNAI1* gene is expressed at low levels in MCF-7 cells (50,51) and is thus characteristic of a gene poised for activation. Therefore, we followed Pol II pausing on *SNAI1* gene during its transcriptional activation. We tested three stimuli that are known to induce *SNAI1 in vitro*. First, we used 17β-estradiol (E2), a natural ligand of estrogen receptor alpha that was previously shown to cause upregulation of *SNAI1* in MCF-7 cells (17). While MCF-7 cells showed an increase in *SNAI1* RNA levels, we observed a modest, but reproducible increase of Pol II pausing signal accompanying activation of *SNAI1* gene (Figure 3A). The small fold-increase in pausing signal is consistent with a transient nature of activation of genes including *SNAI1* by E2 reported previously (17). Importantly, the pause signal did not decrease or change in location during the course of activation by E2. To gain further insight into the fate of Pol II pausing in Snail gene activation, we tested TGF-beta, a cytokine that is a natural inducer of EMT and *SNAI1* (52). However, few *in vitro* models respond to TGF-beta during short time treatment (53) and MCF-7 cells did not. Nevertheless, we found a robust response to TGF-beta and activation of *Snai1* in a classic model of EMT, the mouse NMuMG (transformed, non-cancerous) cells. We note that unlike the human gene, the mouse *Snai1* is not paused in NMuMG cells at basal conditions (Figure 3B). However, during activation, the gene acquired a pausing signature downstream of the TSS in a location that is consistent with pausing in other genes, including human *SNAI1*, before activation (Figures 2 and 3).

Activation of *SNAI1* by E2 is too short-lived (17) to determine the mechanism responsible for the retention in promoter Pol II signal. Therefore, we chose an HDAC inhibitor TSA, which has been previously shown to cause upregulation of *SNAI1* (54). When MCF-7 cells were treated with TSA, *SNAI1* mRNA levels increased within 3 h (3C lower panel). Permanganate signal increased at the same 3-h time point (Figure 3C) without changes in pausing location. ChIP followed by qPCR showed a concomitant increase in Pol II signal (Supplementary Figure S6). *CDH1* gene did not significantly change in expression and, consequently, did not show detectable changes in permanganate reactivity or in Pol II ChIP signal (Figure 3C). Accordingly, the non-paused *SNAI2* gene acquired a pausing signature as it was activated (Figure 3C). Location of permanganate reactivity on *SNAI2* gene, ∼50 nt from the TSS (Figures 3C and Supplementary Figure S8), is similar to the location of Pol II on genes paused before activation. Overall, these observations show that Pol II pausing can be retained—or acquired—during gene activation.

### Pol II pausing does not change during *SNAI1* gene activation

**Figure 4.**
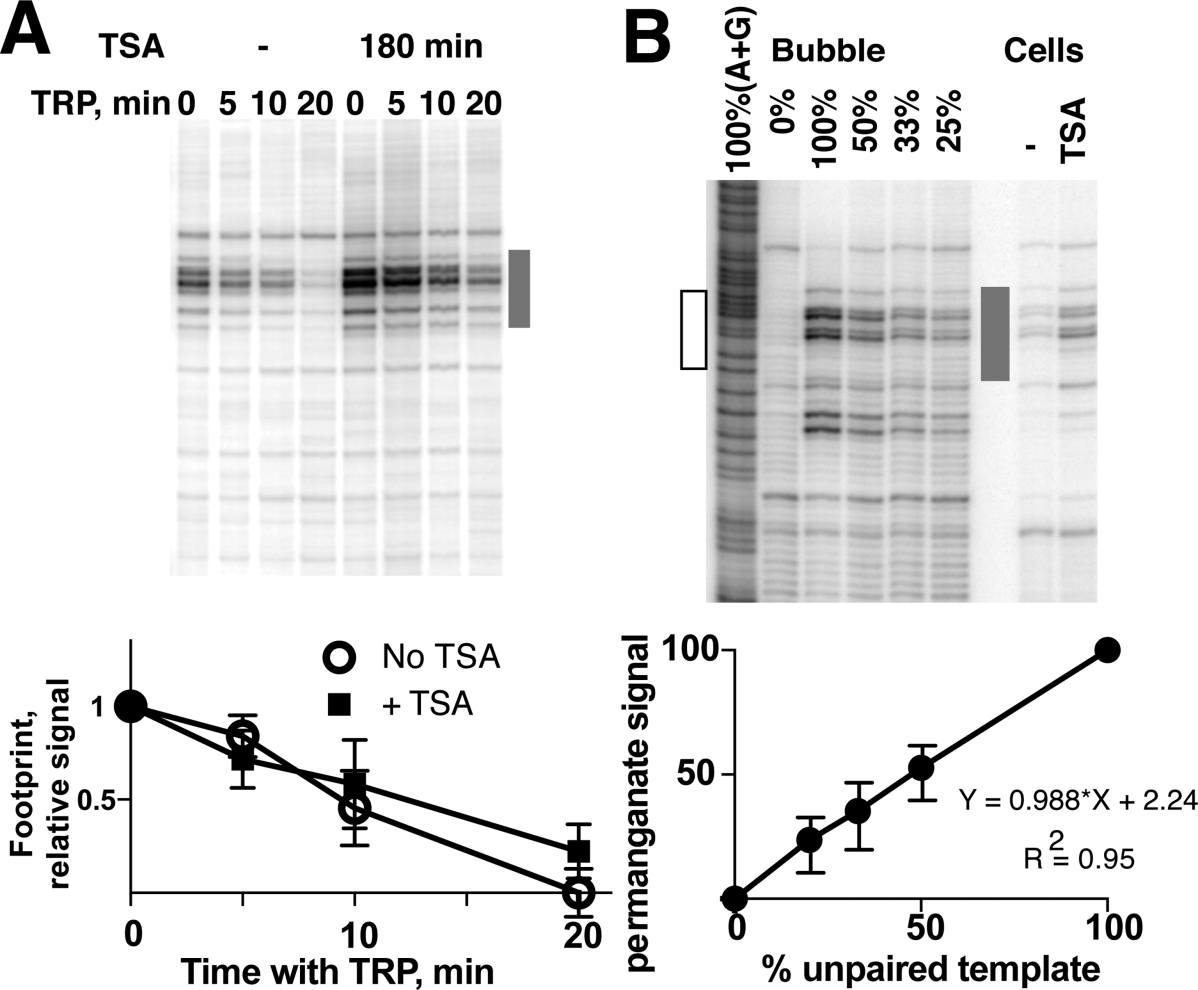
*SNAI1* gene is activated without changes in Pol II pausing duration. (**A**) Sensitivity of paused complexes to triptolide does not change during *SNAI1* activation. Permanganate footprints (top) were done as in Figure 3. (Bottom) Quantitation of permanganate footprints as in (A) represented as fraction of permanganate reactivity compared to a data point without Trp. The values were obtained from three independent experiments. Reactivity of TRP-untreated samples for each time course was normalized to 1. (**B**) Permanganate footprinting of artificial bubble templates done with Snail +100 primer set. A+G ladder was prepared from 100% bubble template. Reactivity of MCF-7 cells, treated and untreated with TSA, is shown for comparison. (Bottom) Quantitation of three independent preparations of bubble templates as in (A), derived from three independent template reconstitution experiments. Reactivity of the full bubble template was normalized to 100%. The hollow rectangle shows the boundaries of the bubble on the A/G ladder.

The promoter Pol II signal can arise either from stable pausing of an individual Pol II molecule or from a rapid turnover of multiple polymerases each with shorter residence time. Therefore, the observed increase in Pol II signal on *SNAI1* gene during activation does not rule out increased polymerase turnover associated with pause release. To determine the mechanism responsible for *SNAI1* gene activation, we sought to determine the duration of Pol II pausing on *SNAI1* gene before and during activation. To do so, we treated cells with triptolide (TRP), a specific inhibitor of TFIIH (55,56). The use of TRP enables one to assess the stability of Pol II pausing complexes (30,41,57). The half-life of paused Pol II on *SNAI1* gene in MCF-7 cells is ∼10 min (Figure 4A), which is consistent with previous observations in *Drosophila* and mouse cells (30,41). At induced conditions (180 min after TSA addition), the duration of Pol II pausing on *SNAI1* gene did not decrease in comparison to the uninduced state (Figure 4A). The observed inhibition of transcription is not due to Pol II protein degradation in the presence of TRP, since levels of Pol II did not change during treatment [Supplementary Figure S9 and (30,41,57)]. Thus, activation of *SNAI1* gene can occur without changes in Pol II pausing.

The observed increase in paused site occupancy is possible only if *SNAI1* gene was not fully occupied by Pol II prior to activation. To confirm this and estimate the extent of the gene occupancy by Pol II, we determined the response of permanganate footprinting signal to the fraction of unpaired DNA. To do that, we measured permanganate reactivity of synthetic DNA molecules based on human *SNAI1* gene sequence and containing a defined fraction of the bubble at the promoter-proximal region of *SNAI1* (see the Materials and Methods section). As shown in Figure 4B, permanganate footprinting of the synthetic bubble templates results in a qualitatively linear response of permanganate signal against the fraction of the bubble template. While we could not determine the exact proportion of unpaired templates in MCF-7 cells due to intrinsic differences in background signals between Pol II complexes *in vivo* and purified templates, the linearity of permanganate signal against the fraction of unpaired DNA implies the commensurate increase in the fraction of occupied *SNAI1* gene in the cell (Figure 4B). The increase in permanganate reactivity in activated cells compared to cells before activation (2.338, SD = 0.1689, *P* = 0.0051, obtained from an independent samples *t*-test using six biological replicates as in Figure 3) indicates that the occupancy of *SNAI1* gene by Pol II in MCF-7 cells is no more than ∼40%, before activation.

### Elevated gene expression is associated with increased pausing signal independent of activation

**Figure 5.**
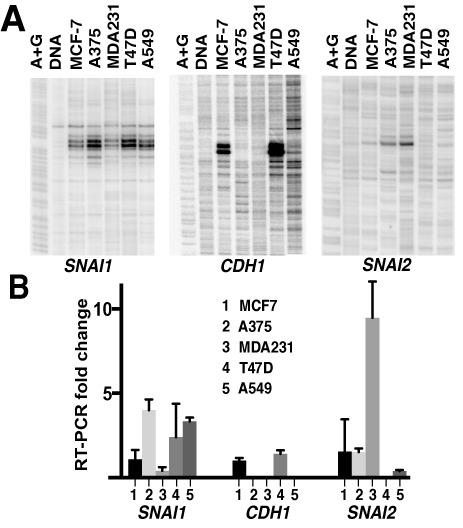
Pol II pausing increase is associated with higher basal levels of transcription independent of activation. (**A**) Pol II pausing signal increases with basal levels of expression. Permanganate footprinting was done on indicated cell lines that were grown in the same media and probed at steady state conditions without stimulation. (**B**) mRNA levels analyzed in the same cell lines as in (A). All values derive from three independent experiments. Data are normalized as Ct difference against GAPDH. For each gene, the mean value for MCF-7 cells is set to 1 and all data points are normalized to this mean value.

Even though Pol II pausing signal increases during Snail gene activation with different stimuli, it is possible that accumulation of Pol II pausing and productive transcription take place at different times during a transcriptional response. Therefore, to determine whether higher Pol II pausing signal is coupled to active transcription, we next sought to observe the same gene in different regulatory environments at steady state conditions. To do so, we compared Pol II pausing signal (Figure 5A) and expression levels (Figure 5B) on *SNAI1, CDH1* and *SNAI2* genes in several cancer cell lines grown in steady-state conditions in the absence of activation. We determined that Pol II pausing signal is higher in cell lines with higher basal expression, shown for *SNAI1, CDH1* and *SNAI2* genes (Figure 5). These results indicate that the increase in Pol II pausing signal with higher expression levels is not limited to gene activation conditions or specific timing during a transcriptional response. Instead, higher pausing signal on more active genes represents a fundamental relationship between Pol II pausing and activity on these genes. The differences between the same gene across these cell lines are likely due to different rates of Pol II initiation supported by the promoter in each cell type, not by the differences in pausing status of these genes. We note that permanganate reactivity profiles of Pol II pausing on each gene are similar among different cell lines (Figure 5), suggesting that the mechanisms that regulate Pol II pausing in these cell lines are similar as well.

### Pol II pausing on the human Hsp70 gene is retained at the same location at the conditions of pause release

**Figure 6.**
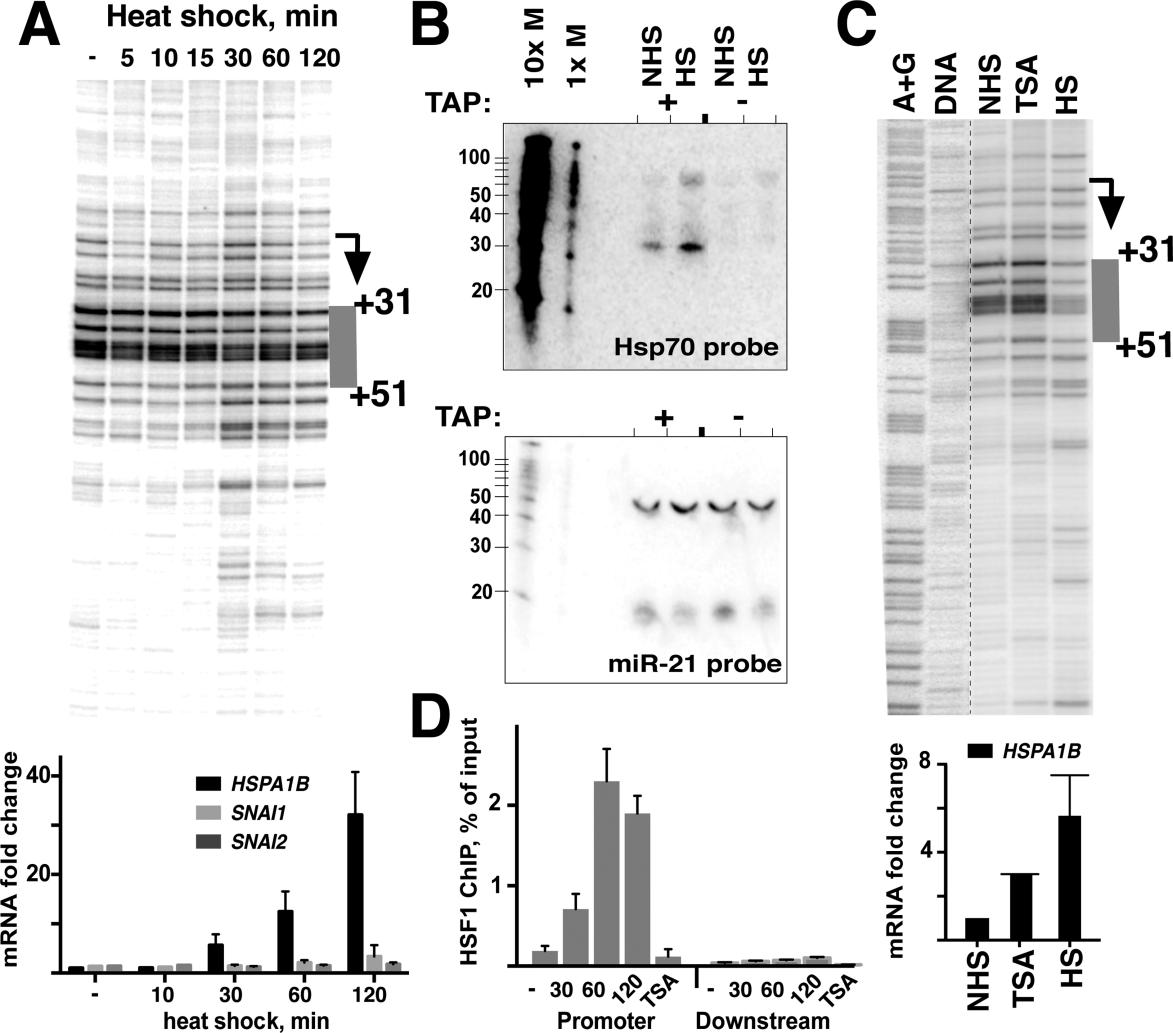
Pol II pausing is retained on *HSPA1B* gene during activation under the conditions of pausing release. (**A**) Permanganate footprinting for the time course of heat shock, shown for *HSPA1B* gene. The region of pausing is highlighted in gray. (**B**) Short RNA northern blot of MCF-7 cells untreated (NHS) or heat shocked for 30 min (HS). The same membrane was re-probed with a control probe against microRNA miR-21, which is not expected to require TAP treatment for crosslinking and does not change with HS. ^32^P-labeled Decade marker is shown on the left of each gel, with the sizes of each band indicated, in nucleotides. (**C**) Activation of *HSPA1B* gene with TSA (3 h) and heat shock (30 min), shown alongside naked DNA and the A/G ladder. (**D**) ChIP combined with qPCR against HSF1 for *HSPA1B* gene for the time course of heat shock induction, with the time of heat shock indicated underneath. The TSA (3 h) sample is shown alongside, as in (C). Numbers represent the percentage of amplifiable DNA recovered after immunoprecipitation with the antibody compared to the input sample.

Having demonstrated that the *SNAI1* gene can be activated without changes in pausing, we next set out to examine a gene where pausing is expected to change, such as a gene regulated through pause release. In *Drosophila*, heat shock causes massive upregulation of heat shock factor (HSF)-responsive genes (25,28,58) that is accompanied by a decrease in pausing duration (28,29). Therefore, we examined *HSPA1B* gene in MCF-7 cells. In addition to being a human paralog of *Drosophila* Hsp70, the archetypal paused gene regulated by pause release, it is also involved in EMT and metastasis [reviewed in (59)]. Before activation, *HSPA1B* gene contained evidence of Pol II pausing (Figure 6A and C). The location of pausing in MCF-7 cells, between positions +31 and +51 downstream from the TSS, is similar to the position of paused Pol II on the same gene in HeLa cells (60) and on Hsp70 gene in *Drosophila* (61–63). Subjecting MCF-7 cells to heat shock led to a rapid increase in *HSPA1B* mRNA levels at the 30-min time point, accompanied by spreading of permanganate reactivity around the original pausing site (Figure 6A). This change in permanganate signal is consistent with increased initiation and downstream escape of polymerase, indicating increased Pol II turnover.

Because of the change in the permanganate reactivity profile at *HSPA1B* gene accompanied by a reduction of signal at the paused site, permanganate footprinting alone could not ascertain the changes in position of Pol II pausing on *HSPA1B* gene during activation. Therefore, we used an alternative approach to directly detect scRNA generated from *HSPA1B* gene by using northern blotting optimized for the detection of scRNAs (41,42). Blotting was done using 5′-phosphate-dependent crosslinking following treatment with the decapping enzyme to detect 5′-capped RNA species. We observed an ∼35-nt predominant scRNA product before activation (Figure 6B). The upstream location of the RNA 3′-end with respect to the transcription bubble indicates that promoter-proximally paused complexes may have undergone backtracking and TFIIS-dependent cleavage, as previously observed on HSP70 gene in *Drosophila* (64). While the levels of the RNA increased during heat shock, the size of the RNA did not change (Figure 6B), indicating that the predominant location of pausing on *HSPA1B* gene remained unchanged during activation. These results indicate that the backtracking of paused complexes continues to take place on *HSPA1B* gene even at the conditions of heat shock activation.

Treatment of cells with TSA also resulted in upregulation of *HSPA1B* gene, but it was accompanied by an increase in permanganate signal (Figure 6C), similar to the *SNAI1* gene (Figure 3C) and consistent with upregulation through transcription initiation. Unlike activation with heat shock, however, TSA caused no increase in the binding of HSF1 factor in the promoter region, suggesting that the two stimuli activate *HSPA1B* gene through different mechanisms (Figure 6D). Taken together, the data indicate that Pol II pausing is retained during heat shock gene activation and remains at the same location even at the conditions of pause release.

## DISCUSSION

We demonstrate that genes can retain Pol II pausing during activation even at the conditions of pause release. Stable pausing has been previously suggested to serve as a platform for integration of regulatory inputs (41,65). The main conceptual outcome of our current work is that Pol II pausing continues to provide a stable platform during transcriptional responses.

scRNA sequencing positions Pol II in MCF-7 cells on many genes with the predominant distance of 33–35 nucleotides downstream of the TSS (Figure 1). Using paired-end ChIP-sequencing in the same cells, however, we observed two promoter-proximal peaks, which had been recently reported through re-analysis of earlier Pol II ChIP-sequencing data (Figure 1) (45). We suggest that these ChIP-sequencing peaks do not correspond to actual locations of Pol II at promoters and do not indicate the existence of stable closed complexes, for the following reasons. First, we do not see stable open complexes at the start sites of genes that we individually analyzed by permanganate footprinting (Figure 2). Second, the 3′-ends of scRNAs in MCF-7 cells as well as high-resolution run-on analysis in a different cell line (Figure 1G) (44) place Pol II active sites across the genome in the middle between these two peaks (Figure 1). While the precise origins of these peaks remain to be determined, we suggest that they arise because of sonication as a method of choice for fragmentation of chromatin. We speculate that breakage of DNA by sonication may be disfavored in the regions adjacent to the paused Pol II complex, leading to the bias in shearing and DNA fragment locations. Pol II, therefore, pauses in one predominant location in the promoter proximal regions of many genes. We also note that the TSSs defined by our scRNA sequencing and recently published GRO-CAP approaches are remarkably alike (Figure 1 and Supplementary Figure S10). That the TSSs are similar even between different human cell lines indicates that despite the pervasive nature of transcription throughout the genome (66), the process of initiation at the nucleotide level is highly robust. Accordingly, the 3′-ends of scRNAs in MCF-7 cells correspond well to the locations of Pol II pausing determined by permanganate footprinting here and by high-resolution GRO-sequencing approach (PRO-seq) in a different cell line (44) (Figure 1 and Supplementary Figure S10). The paired end scRNA sequencing approach that we report here defines the start sites of transcription and sites of Pol II pausing simultaneously, combining many of the benefits of the GRO-CAP and PRO-Seq analyses (44). Further analysis of early transcription combining run-on and free RNA-based methods will shed light into the mechanisms and global dynamics of pausing during stimulus response.

Using several EMT-related genes for detailed analysis, we show that gene activation can take place without changes in pausing. In particular, our findings suggest that promoter enrichment previously stipulated to take place on active genes (15) or activated genes (33) is due to retention of stable Pol II pausing at the same location. Conversely, while genes such as mouse *Snai1* and human *SNAI2* may be classified as non-paused based on low Pol II abundance near their promoters, the genes only appear so because of low activity of the promoter to drive transcription initiation and, in fact, support pausing at the conventional location once their promoters become sufficiently active (Figure 3). Mechanistically, it is the availability of the negative elongation factor (NELF) during each transcription initiation event that matters for the establishment of pausing status (23,67–68). Therefore, we envision that a different regulatory environment, as a result of cell differentiation, environmental influence or a drug exposure, can alter the availability of NELF on the gene, perhaps through epigenetic mechanisms, and thus stably change its pausing state. We note that because human *SNAI2* gene does not contain a CpG island around its promoter (Supplementary Figure S11), the presence of an overlapping CpG island is not strictly required for pausing, suggesting that mechanisms other than DNA methylation directly control pausing. Consequently, while TSA and other broad-spectrum epigenetic drugs are used to target multiple types of cancers (12,69), the mechanisms of their action and sometimes their effects remain uncertain. We suggest that the ‘hidden paused’ genes, exemplified by *SNAI2*, contribute to the uncertainty in drug responses because they might not be distinguishable from non-paused genes using traditional biomarkers relying on the detection of mRNA. Further, our finding that TSA upregulates the heat shock *HSP70* gene independent of HSF1 factor suggests that TSA, and HDAC inhibitors in general, can affect genes through bypassing their cognate mechanisms. Understanding how broad-spectrum epigenetic drugs affect gene transcription will help anticipate their effects on different cell types and rationalize their use for combination therapies of cancer (70) and neurological disorders (71).

Heat shock activation of human *HSPA1B* gene involves pause release that takes place without changes in pausing location (Figure 6). Because *HSPA1B* gene displays a highly robust transcriptional response to heat shock, it is likely that other genes undergoing pause release in response to their cognate stimuli can retain pausing at the same location as well. Early transcription elongation is a dynamic process and depletion of factors involved in the regulation of pausing or mutation of Pol II has been shown to lead to a shift in the location of paused Pol II (48). That the location of pausing on *HSPA1B* gene did not change during activation in MCF-7 cells suggests that all factors necessary for pause establishment and release are available at the promoter at the conditions of faster pausing turnover. The mechanisms that enable higher turnover without a shift in location may include requirement for a particular sequence of events, such as binding or phosphorylation of NELF prior to pause release mediated by P-TEFb (72), interaction of paused Pol II with a defined set of factors (41) or recruitment of multiple functions through protein complexes such as Super Elongation Complex or the Integrator (73,74). While the interplay between pausing establishment and release remains to be fully understood, our data show that Pol II pausing remains the slowest step on heat shock gene in human cells even at the conditions of pause release.

Our findings suggest that the paused *SNAI1* gene is not fully occupied by Pol II in MCF-7 cells. Given that the *SNAI1* is well within the top 20% of genes based on promoter-proximal Pol II enrichment in MCF-7 cells (Supplementary Figure S12), it is likely that many if not most of the paused genes are not fully occupied in MCF-7 cells, and probably in other mammalian systems as well. The incomplete fractional occupancy of genes in a population may reflect both the stable heterogeneity of cell populations, as observed in a number of systems including stem cells [reviewed in (75)], and dynamic changes in the same cell (76,77). It follows that transcription initiation, not pausing, might be the limiting step for the overall transcription output even for highly paused genes. We note that the incomplete fractional occupancy by Pol II allows genes to be poised for activation through pause release and at the same time supports activation through recruitment of additional Pol II to unbound gene copies. Overall, we propose that the increase of pausing signal represents the default outcome of gene activation that enables context-specific regulatory inputs including pause release. Further analysis of pausing in different systems will uncover its role both in poising genes prior to activation and in regulating transcriptional responses to stimuli.

## SUPPLEMENTARY DATA

Supplementary Data are available at NAR Online.

SUPPLEMENTARY DATA
