# Supplementary material for: RNA polymerase II pausing can be retained or acquired during activation of genes involved in the epithelial to mesenchymal transition
Source: Nucleic Acids Res. 2015 Mar 27;43(8):3938–49. doi: 10.1093/nar/gkv263 (PMC4417172; doi:10.1093/nar/gkv263)
Supplement: SUPPLEMENTARY DATA [file supp_43_8_3938__index.html]

RNA polymerase II pausing can be retained or acquired during activation of genes involved in the epithelial to mesenchymal transition — SUPPLEMENTARY DATA 

# RNA polymerase II pausing can be retained or acquired during activation of genes involved in the epithelial to mesenchymal transition

## SUPPLEMENTARY DATA

**Files in this Data Supplement:**

- SUPPLEMENTARY DATA
