# Supplementary material for: RNA polymerase II pausing can be retained or acquired during activation of genes involved in the epithelial to mesenchymal transition
Source: Nucleic Acids Res. 2015 Mar 27;43(8):3938–49. doi: 10.1093/nar/gkv263 (PMC4417172; doi:10.1093/nar/gkv263)
Supplement: SUPPLEMENTARY DATA [file supp_gkv263_nar-00490-x-2015-File008.pdf]

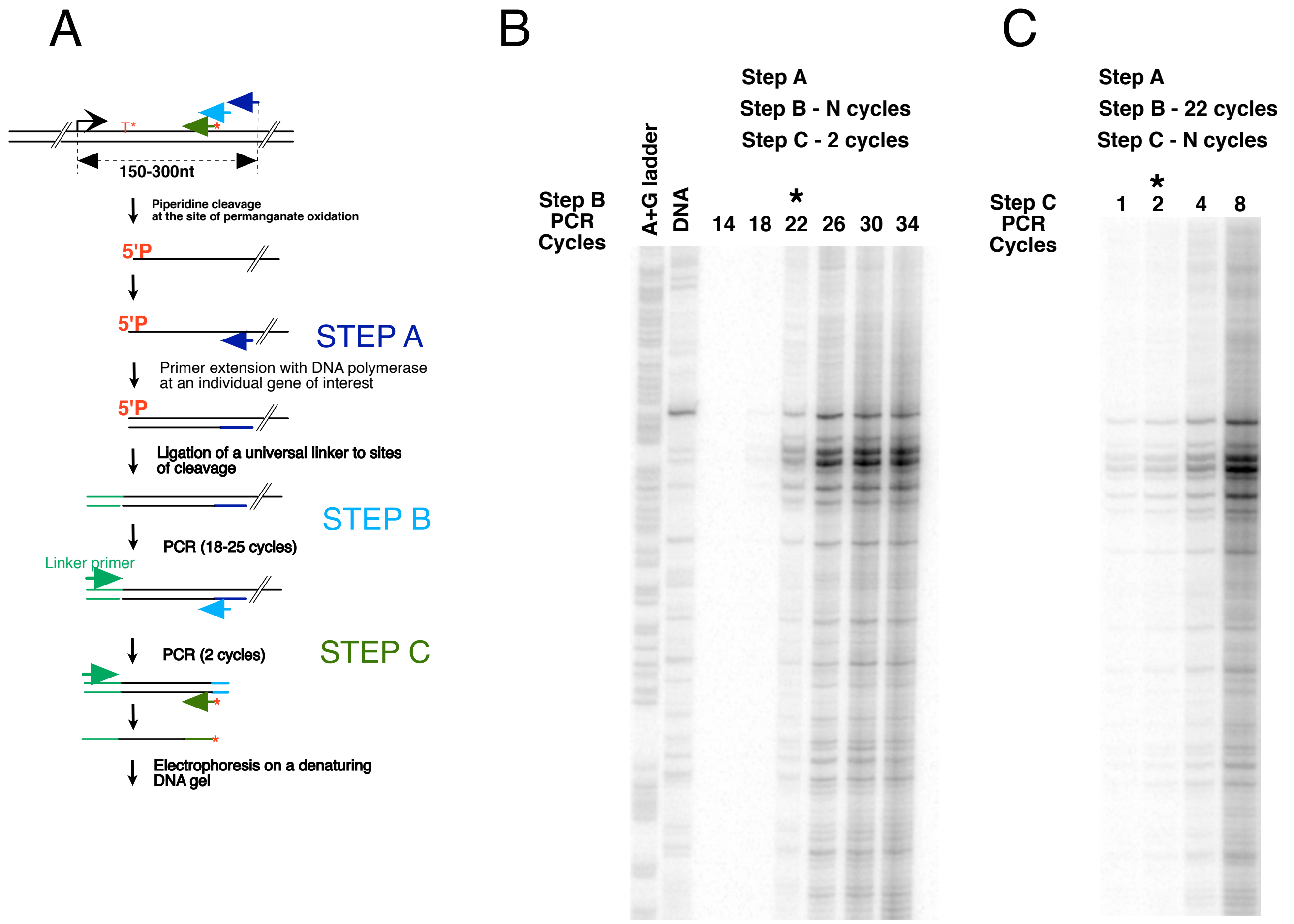
A
T*
150-300nt
Piperidine cleavage
at the site of permanganate oxidation
5'P
5'P
STEP A
Primer extension with DNA polymerase
at an individual gene of interest
5'P
Ligation of a universal linker to sites
of cleavage
STEP B
PCR (18-25 cycles)
Linker primer
PCR (2 cycles)
STEP C
Electrophoresis on a denaturing
DNA gel
B
Step A
Step B - N cycles
Step C - 2 cycles
Step B
PCR
Cycles
A+G ladder
DNA
14
18
*
22
26
30
34
C
Step A
Step B - 22 cycles
Step C - N cycles
Step C
PCR
Cycles
1
*
2
4
8

Figure S1

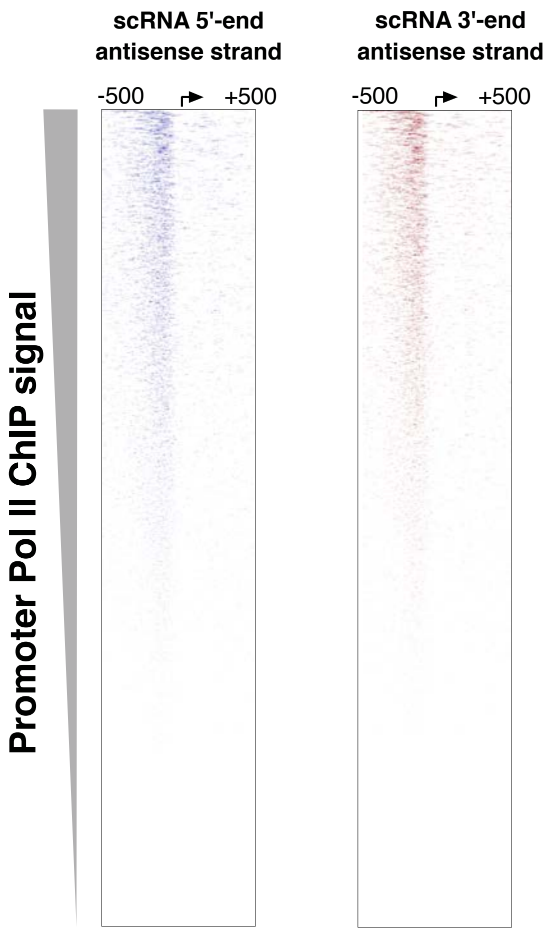
scRNA 5'-end
antisense strand
-500
+500
scRNA 3'-end
antisense strand
-500
+500
Promoter Pol II ChIP signal

Figure S2

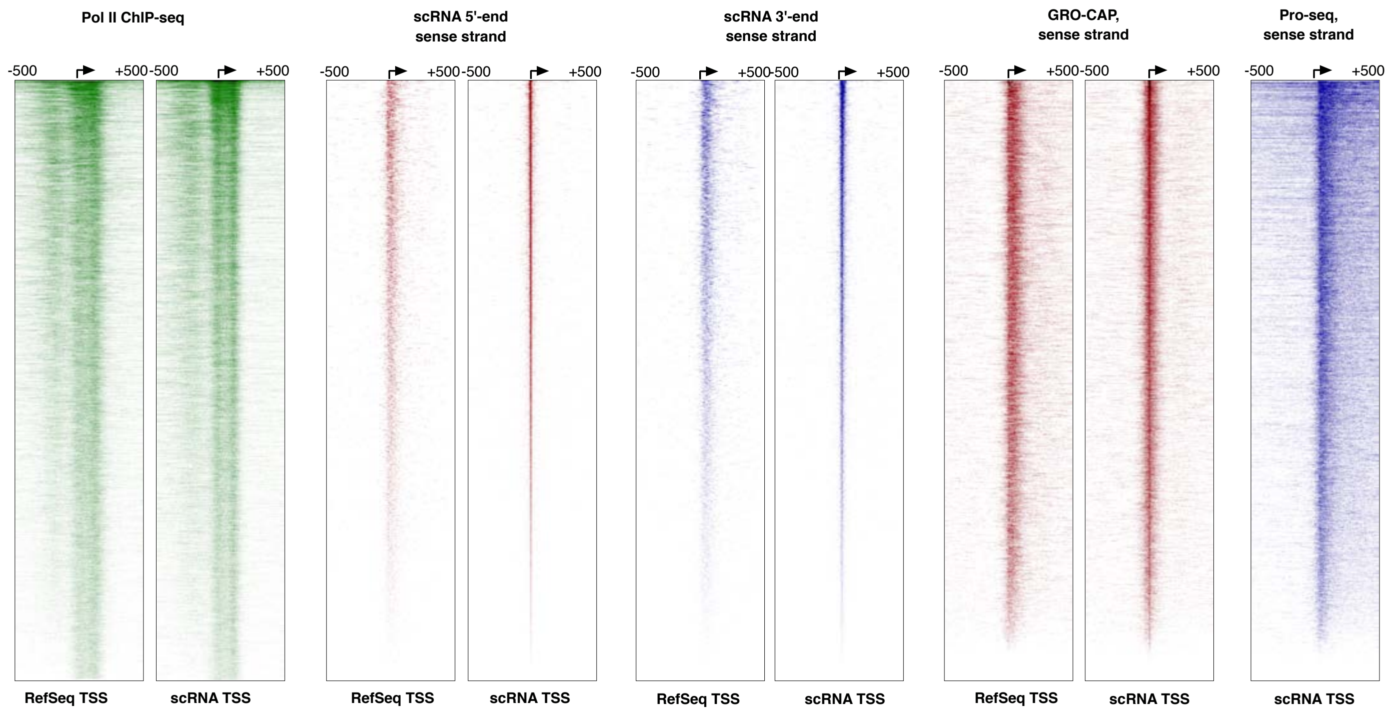
Pol II ChIP-seq
scRNA 5'-end
sense strand
scRNA 3'-end
sense strand
GRO-CAP,
sense strand
Pro-seq,
sense strand
-500
+500
RefSeq TSS
scRNA TSS

Figure S3

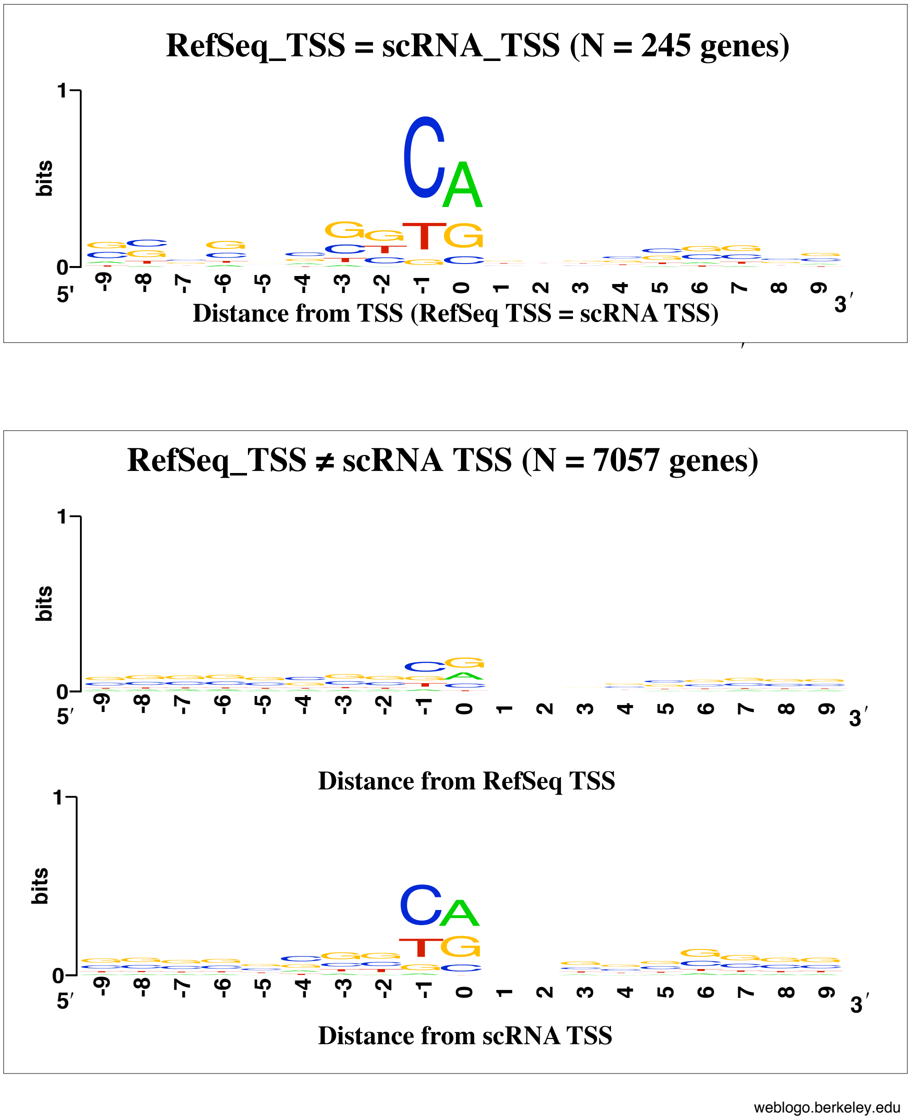
RefSeq_TSS = scRNA_TSS (N = 245 genes)
bits
Distance from TSS (RefSeq TSS = scRNA TSS)
RefSeq_TSS ≠ scRNA TSS (N = 7057 genes)
bits
Distance from RefSeq TSS
bits
Distance from scRNA TSS
weblogo.berkeley.edu

Figure S4

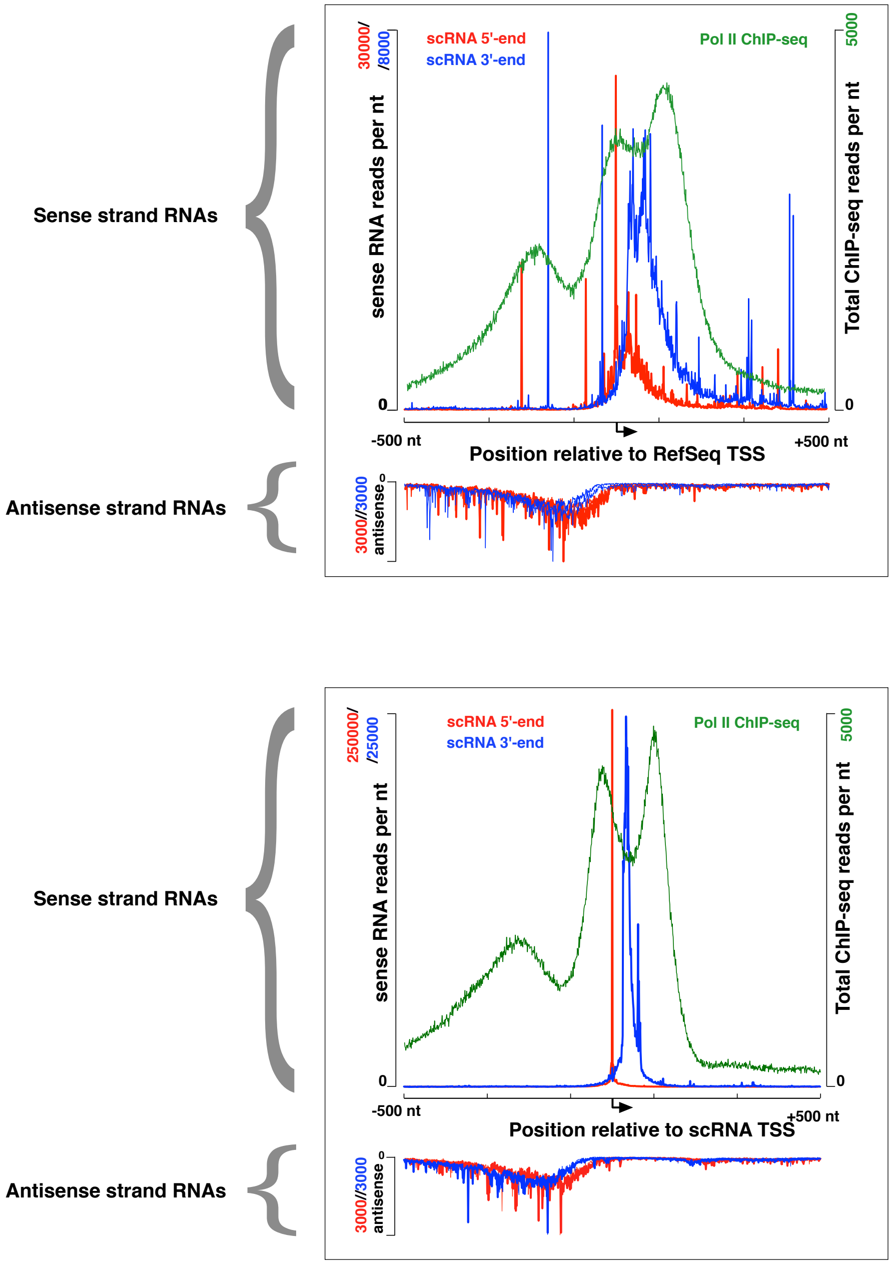
Sense strand RNAs
Antisense strand RNAs
scRNA 5'-end
scRNA 3'-end
Pol II ChIP-seq
30000/
/8000
5000
sense RNA reads per nt
Total ChIP-seq reads per nt
0
0
-500 nt
+500 nt
Position relative to RefSeq TSS
3000//3000
antisense
0
Sense strand RNAs
Antisense strand RNAs
scRNA 5'-end
scRNA 3'-end
Pol II ChIP-seq
250000/
/25000
5000
sense RNA reads per nt
Total ChIP-seq reads per nt
0
0
-500 nt
+500 nt
Position relative to scRNA TSS
3000//3000
antisense
0

Figure S5

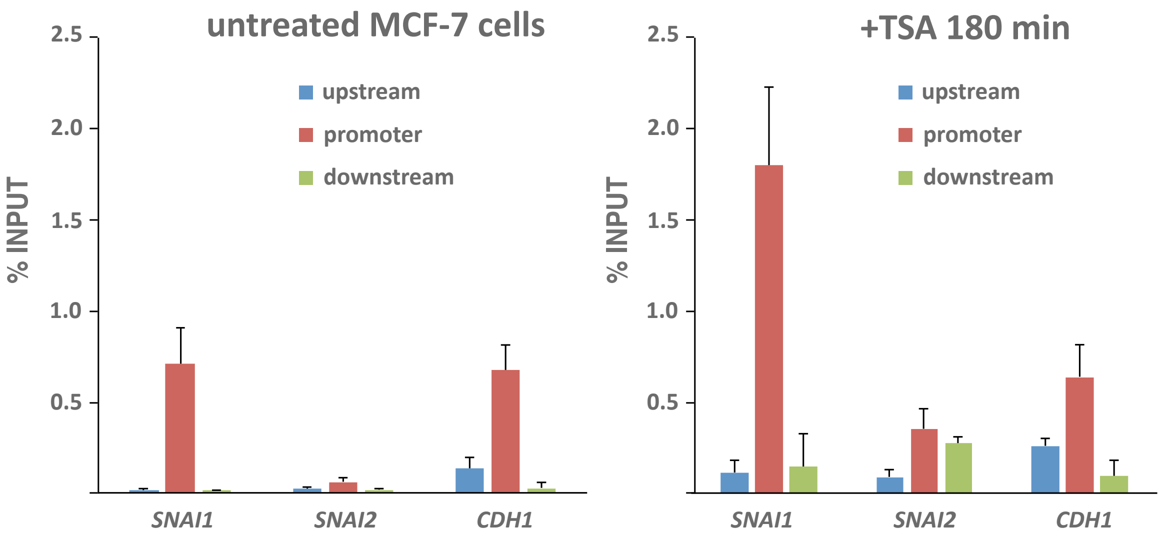
untreated MCF-7 cells
+TSA 180 min
upstream
promoter
downstream
% INPUT
2.5
2.0
1.5
1.0
0.5
SNAI1
SNAI2
CDH1

Figure S6

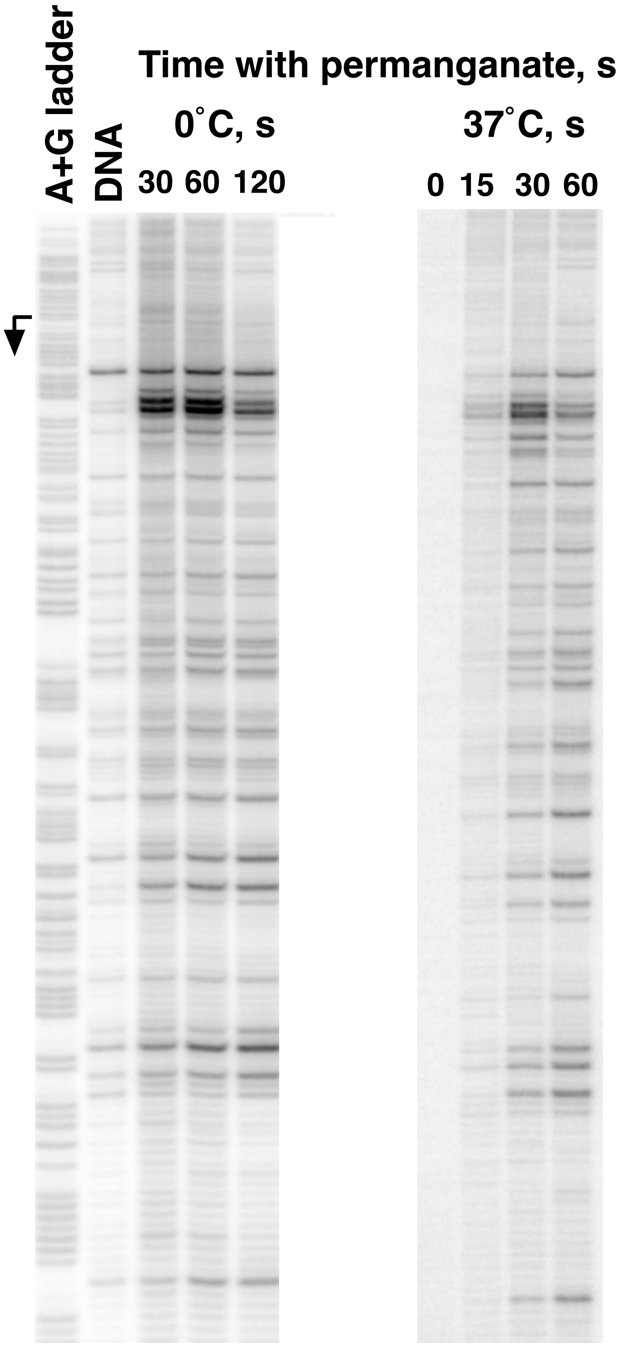

A+G ladder
DNA
Time with permanganate, s
0˚C, s
30 60 120
37˚C, s
0 15 30 60

Figure S7

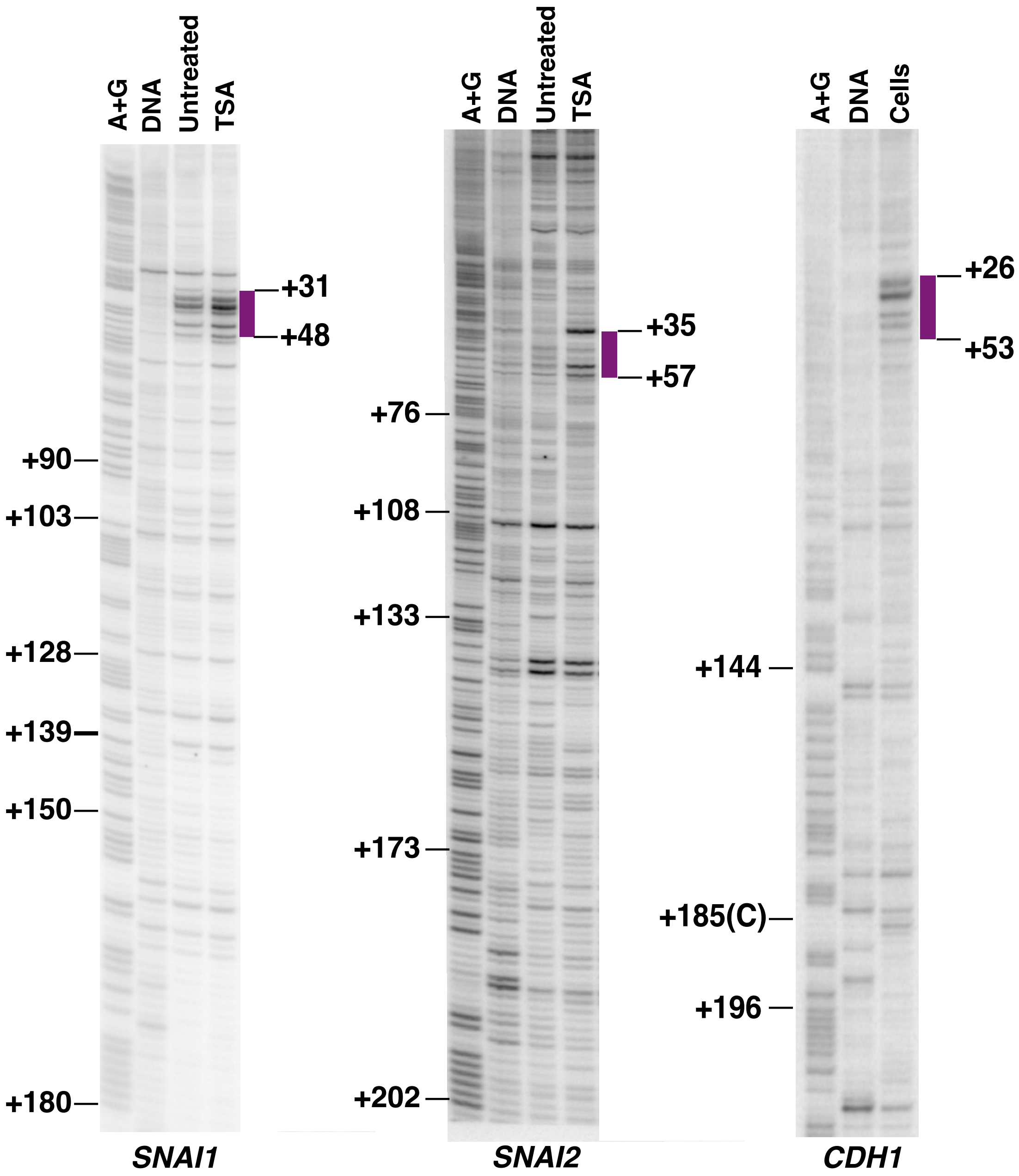
A+G
DNA
Untreated
TSA
+31
+48
+90
+103
+128
+139
+150
+180
SNAI1
A+G
DNA
Untreated
TSA
+35
+57
+76
+108
+133
+173
+202
SNAI2
A+G
DNA
Cells
+26
+53
+144
+185(C)
+196
CDH1

Figure S8

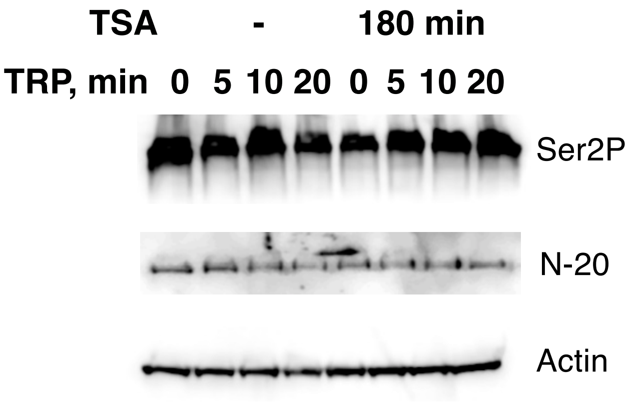

Figure S9

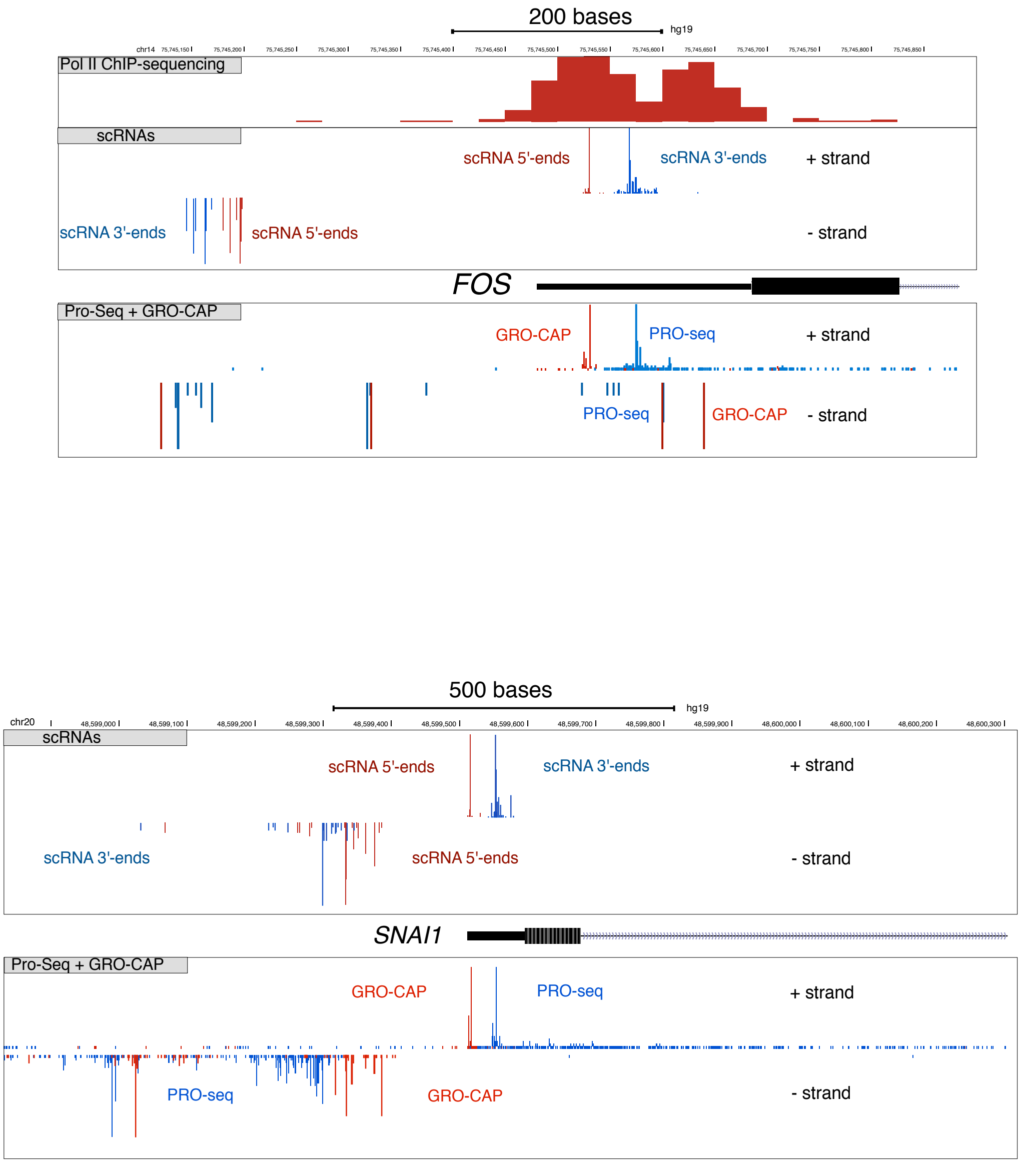
200 bases
hg19
chr14
Pol II ChIP-sequencing
scRNAs
scRNA 5'-ends
scRNA 3'-ends
+ strand
scRNA 3'-ends
scRNA 5'-ends
- strand
FOS
Pro-Seq + GRO-CAP
GRO-CAP
PRO-seq
+ strand
PRO-seq
GRO-CAP
- strand
500 bases
hg19
chr20
scRNAs
scRNA 5'-ends
scRNA 3'-ends
+ strand
scRNA 3'-ends
scRNA 5'-ends
- strand
SNAI1
Pro-Seq + GRO-CAP
GRO-CAP
PRO-seq
+ strand
PRO-seq
GRO-CAP
- strand

Figure S10

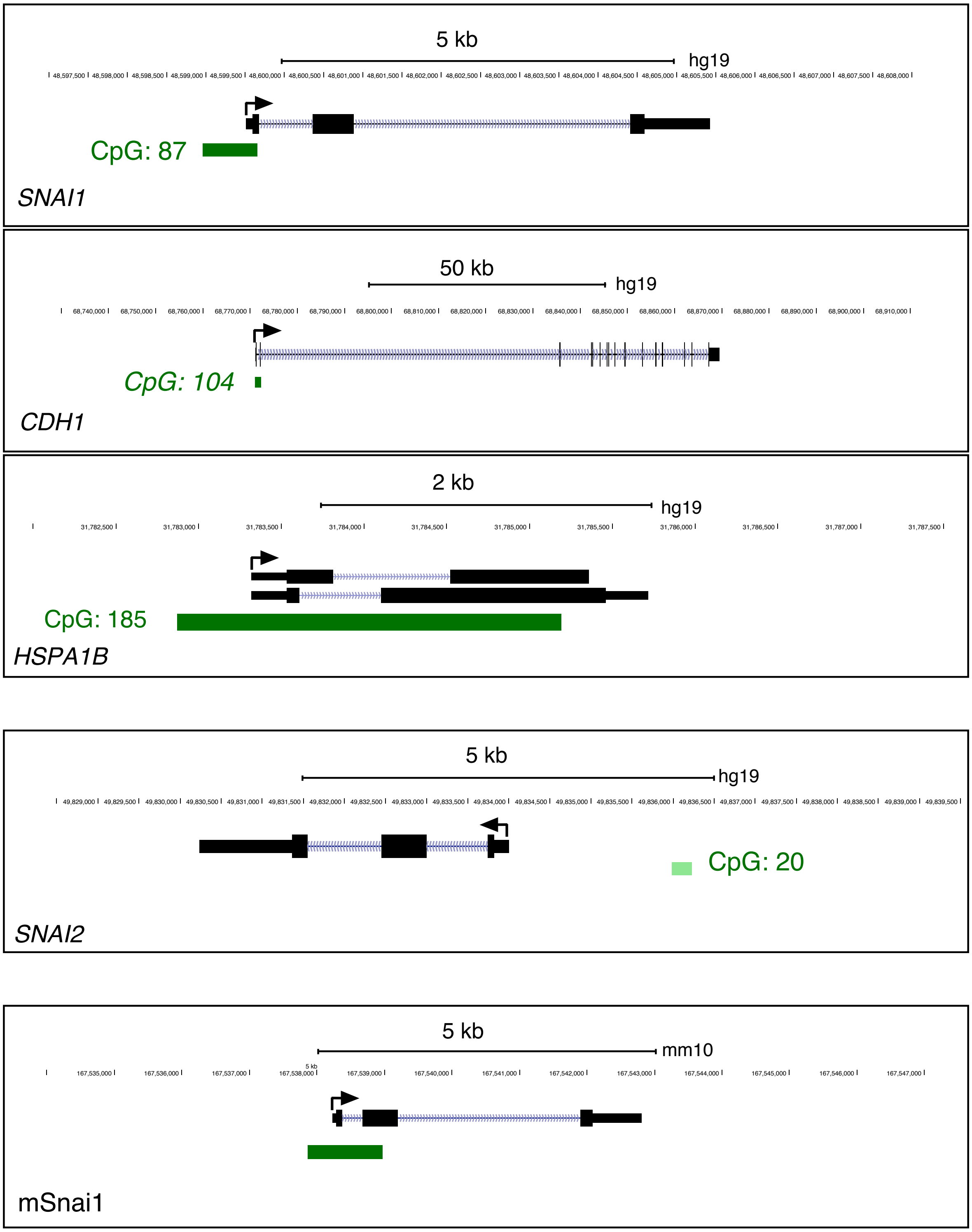
5 kb
hg19
CpG: 87
SNAI1
50 kb
hg19
CpG: 104
CDH1
2 kb
hg19
CpG: 185
HSPA1B
5 kb
hg19
CpG: 20
SNAI2
5 kb
mm10
mSnai1

Figure S11

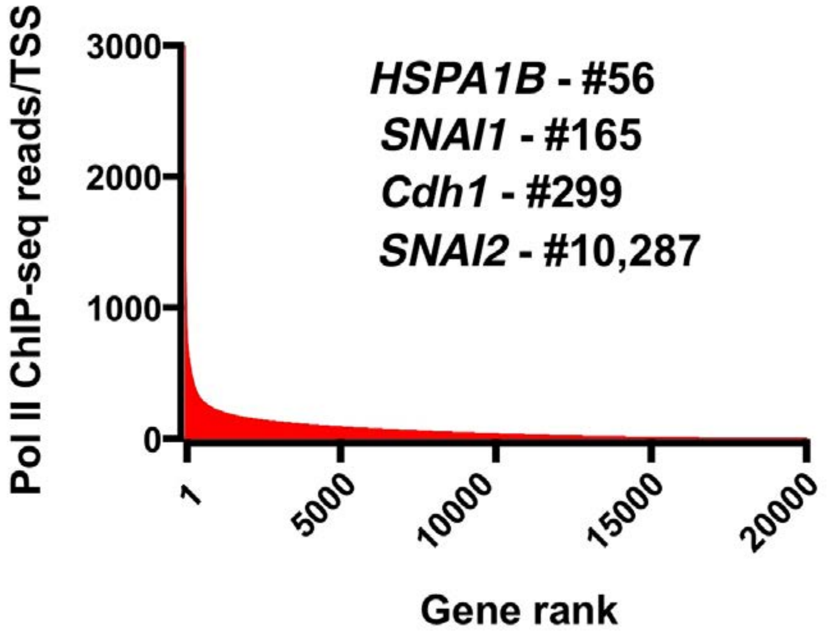
HSPA1B - #56
SNAI1 - #165
Cdh1 - #299
SNAI2 - #10,287
Pol II ChIP-seq reads/TSS
3000
2000
1000
0
1
5000
10000
15000
20000
Gene rank

Figure S12

SUPPLEMENTARY MATERIAL.

Supplementary Figure Legends.

Figure S1. Optimization of permanganate footprinting in MCF-7 cells. A. A scheme of LM-PCR permanganate footprinting beginning with DNA extracted from permanganate-treated material. Positions of primers A, B, C used in Ligation-Mediated PCR protocol are indicated with arrows of different colors. The preferred distance of primers from the TSS is ~150-300 nucleotides. We note that the A, B, or C primers do not need to overlap. **B.** The permanganate footprint does not change within a broad range of PCR cycles. Optimization of PCR cycles during step B (amplification) on *SNAI1* gene. Two hundred nanogram of MCF-7 DNA prepared from cells treated with permanganate was used for each reaction. The number of PCR cycles is shown above the gel. **C.** Optimization of PCR cycles during step C (with radioactively labelled primer) on *SNAI1* gene. The conditions reflecting the number of cycles used in experiments in the main text are marked with asterisks.

Figure S2. Pol II pausing in divergent transcription. scRNA heatmaps show antisense transcription for the same genes as in Figure 1. Genes are sorted based on promoter-proximal Pol II enrichment from the ChIP-sequencing data as in Figure 1B. 5'-ends of antisense scRNA are shown in red and 3'-ends in blue.

Figure S3. scRNAs represent transcription start sites. Heatmaps are shown as in Figure 1, except that in addition to centering on RefSeq TSSs, the coordinates are also centered based on the positions of TSSs defined based on scRNAs, shown for ChIP-seq, 5', and 3' RNA seq. The rightmost three plots show GRO-CAP and Pro-seq tracks for K562 cell line from (42).

Figure S4. Enrichment of sequence motifs around the TSSs. The top panel shows motif enrichment around the TSS for genes where RefSeq TSSs matched exactly the scRNA TSSs. The bottom panel shows motif enrichment for genes where the RefSeq TSS differs from scRNA-defined TSS. Defining TSSs through scRNAs increases the enrichment of the initiator motif, suggesting that scRNAs better reflect the TSSs of mRNA transcription. Motif enrichment was generated with Weblogo.

Figure S5. Metagene profiles of scRNAs. The plots correspond to heatmaps shown in Figure 1 and Figure S3, except that divergent (antisense) transcription is shown to scale in addition to the sense transcription. Plots are centered against RefSeq annotated TSS (top panel) and against scRNA TSSs (bottom panels). ChIP-sequencing traces for Pol II are shown for reference.

Figure S6. Promoter-proximal Pol II enrichment on individual genes. N-20 Pol II ChIP was combined with qPCR with primers specific to the indicated genes in MCF-7 cells untreated or treated with TSA for 3h. The sequences of primers are given below. Values in the Y-axis represent the percentage of PCR-amplifiable DNA recovered in ChIP samples compared to input DNA, combined from three independent biological replicates.

Figure S7. Absence of stable open complexes on *SNAI1* gene in MCF-7 cells. Titration of the timing of permanganate treatment. Cells were treated with permanganate for the times indicated. In the right panel, cells were treated directly in media at 37°C, within less than 30s of being withdrawn from CO_2 atmosphere incubator. Left panel shows footprinting performed on ice using a standard procedure. The position for the TSS of *SNAI1* gene is shown with an arrow.

Figure S8. Permanganate footprinting in promoter-proximal regions downstream of the pausing site. Permanganate footprints as in Figure 2 are shown for more downstream regions, with positions relative to the annotated TSSs shown next to each gel.

Figure S9. Integrity of Pol II during Trp treatment. Western blot with anti Pol II Ser-2P antibody (Millipore, Clone 3E10) and N-20 antibody for lysates prepared from cells used for a time course of TRP treatment. Western blot of the same samples with anti β-actin antibody is also shown as loading control.

Figure S10. Genome browser view of example human genes. The UCSC browser derived tracks are based on our ChIP-sequencing and scRNA sequencing data for MCF-7 cells, and for Pro-Seq and GRO-CAP data for K562 cells (42). The *top panel* shows FOS gene. The database TSS is 50 nt upstream of the scRNA TSS defined by us in MCF-7 cells. The Gro-CAP sequencing of K562 cell line points to the same TSS as that defined by scRNAs. *Bottom panel.* RNA tracks are shown for

SNAI1 gene. The annotated TSS is 4 nt upstream of the TSS defined by scRNAs by us in MCF-7 cells and using GRO-CAP in K562 cells (42).

Figure S11. CpG islands around promoters of genes analyzed in this work. Genome browser views of individual genes with CpG island tracks from UCSC browser shown for human genes based on hg19 genome and mouse *Snai1* based on mm10 genome annotation.

Figure S12. *SNAI1* gene is among the most highly paused in MCF-7 cells. The plot shows counts of ChIP-sequencing hits within promoter-proximal regions (the interval between -100 to +200 nt from the annotated TSS) of the first 20,000 genes, based on list of all RefSeq genes, sorted based on Pol II enrichment in the same interval. Y-axis shows the number of sequencing reads within an interval in a given gene. Numerical ranks of genes individually analysed in this study are shown above the plots.

SUPPLEMENTARY METHODS

ChIP-sequencing.

ChIP-sequencing paired end data files (fastq, 75-nt reads) were aligned against hg19 reference genome with *bowtie* using 75-nt seed size (the entire length of the read) with two allowable mismatches. Reads with non-unique matches and read pairs with the distance exceeding 700 nt between the R1 and R2 matching coordinates were discarded. Bedgraph files were made by returning the center point positions for each mapped read pair. The read center positions were then combined in 25-nt non-overlapping bins for visualization in UCSC browser, in 10-nt bins for heatmap generation and in 1-nt bins (unbinned) for metagene plots. Replicates were normalized based on the number of uniquely mappable reads in each sample and combined into a bedgraph file. The number of uniquely mappable reads was 13438480 + 14715640 = 28,154,120 for untreated MCF-7 cells. Pol II enrichment at the promoters was calculated by summing up the mapped read pairs within the interval from -100 to +200 from each annotated TSS.

scRNA-sequencing.

scRNA sequencing paired end data files (fastq, 50-nt reads) were aligned against hg19 reference genome with *bowtie* using 26-nt seed length after 24-nt nucleotide trimming from the 3'-end of each read, with two allowable mismatches. The number of uniquely mappable reads was 1,610,543 + 1,837,112 + 4,980,050 = 8,427,707 for untreated MCF-7 cells for each of the three replicates. Bedgraph files were generated, separately for each strand, by returning a position of the first nucleotide of R1 reads, which were designated as RNA 5-ends, and of the first nucleotide of R2 reads, which were designated as RNA 3'-ends. Bedgraph files were generated separately from R1 and R2 files using a custom script (available upon request). Distributions of sRNA lengths were made for each read pair based on the distance between R1 and R2 coordinates in each matching pair of sequencing reads.

ScRNA TSS annotation.

The RefSeq gene list containing 50,064 genes was trimmed down to remove matches to chrM as well as MIR, SNORD, SNORA, Y_RNA, TRNA, rRNA, snoRNA, snRNA hits. Transcripts annotated as - AF(<numeric>), AJ(<numeric>), AK(<numeric>), AL(<numeric>), AX(<numeric>), AY(<numeric>), BC(<numeric>) were also removed. Of the remaining genes, duplicate isoforms with the same name were resolved by removing all isoforms except the one with highest Pol II ChIP-sequencing signal enrichment within -100 to +200 nt from the annotated TSS. The resultant list generated 24,441 genes. The top 7302 genes (the top 30% of the filtered gene list after manually removing additional snall noncoding RNA genes such as DM119512) based on Pol II signal enrichment within -100 to +200nt from the annotated TSSs were used to generate heatmaps and metagene plots. The position within -500 to +500 nt region from the annotated TSS in the sense orientation with the highest number of mapped reads was defined as the scRNA TSS. Pro-Seq and Gro-cap data were obtained from GEO GSM1480321 and GSM1480327 and analysed the same way as scRNAs, except that the

number of nucleotides trimmed from the end of each sequence read was adjusted according to the length of the sequences in fastq files.

PRIMER SEQUENCES.

ChIP Primers

Primer name	Sequence
SNAI1-upstream-F	ATAATTCTTCACTTCCTCTGGGAA
SNAI1-upstream-R	TTCTGGTCCAGTGAGGGAGA
SNAI1-promoter-F	AGTGGTTCTTCTGCGCTACTGCT
SNAI1-promoter-R	CGCTGTAGTTAGGCTTCCGATTG
SNAI1-downstream-F	TGTTCCAGTCACAGCTGCTG
SNAI1-downstream-R	GACGCCTTCATGGTACTCCT
SNAI2-upstream-F	AAGCCAAGAGGTAATTATTTGGTC
SNAI2-upstream-R	CTCTGGTGTTAATGAGAGCCTAT
SNAI2-promoter-F	CTCTCAGCTGTGATTGGATCTT
SNAI2-promoter-R	GTCACCCGGCTCCTTTAC
SNAI2-downstream-F	CATGCACTAAGTCTCCTTTCCT
SNAI2-downstream-R	TCCATGTTACGTATGGGCTATTT
CDH1-upstream-F	CTTTCTGATCCCAGGTCTTAGTG
CDH1-upstream-R	TAGGGTCTAGGTGGGTTATGG
CDH1- promoter –F (77)	GGCCGGCAGGTGAAC
CDH1- promoter –R (77)	GGGCTGGAGTCTGAACTGAC
CDH1-downstream-F	CCTGCCTGGTTGTTGACTAT
CDH1- downstream -R	CTACAGTGCTGAGGGCAATAA
HSP70-promoter-F	GTGATTGGCTCAGAAGGGAAA
HSP70-promoter-R	TCCTCAGGCTAGCCGTTATC
HSP70-downstream-F	CGACGACGGCATCTTCGAGGTGAAGGCC
HSP70-downstream-R	GCACCCTGGAGCCCGTGGAGAAGGCTCTGC

Permanganate primers

Primer name	Sequence
hCDH1+272A	TTTCTTGGAAGAAGGGAAG
hCDH1+272B	TTCTTGGAAGAAGGGAAGC
hCDH1+272C	AGAAGGGAAGCGGTGACGAC
hCdh1+185C	ggatccggggtacCTGCAGCA
hSnail+210A	CAATGGTCCACAAAACATC
hSnail+210B	ACATCCTGTGACTCGATCCT
hSnail+210C	TGTCTCCCCCAAACCTCCTG
hSnail+100A	TGTAGTTAGGCTTCCGATT
hSnail+100B	CTTCCTGACGAGGAAAGAG
hSnail+100C	AGGAAAGAGCGCGGCATAGT
Slug+225A	TTTGCAAAGCTCTAGATACG
hSlug+225B	TGTCCAGTTCGCTGTAGTTT
hSlug+225C	GGCTTTTTGGAGGCGTTGAA
hHSPA1B+158A	GGTTCCCTGCTCTCTGTC
hHSPA1B+158B	GTTCCCTGCTCTCTGTCG
hHSPA1B+158C	ACTGGATCCGCGAGAAGAGC
Linker A’	GCGGTGATCGTCGAGATCTGAATTC
Linker B	GAATTCAGATC

Northern probes:

Primer name	Sequence
hsa-miR-21*.mi.1	ACAGCCCATCGACTGGTGTTG/3STArFire/
hLHspA1B+37	TAGTGGACTGTCGCAGCAGCTCC/3STArFire/

Synthetic template primers:

Primer name	Sequence
Upstream_WT-Top strand	CGGAGTACTTAAGGGAGTTGGCGGCGCTGCTGCATTCATTGCGCCGCGGCACGGCCTAGCGAGTGGTTCTTCTGCGCTACTGCTGCGCGAATCGGCGACCCCAGTGCCTCGACCACTATGCCGCGCTCTTTCCTCGTCAGGAAGCCCTCCGACCCCAATCGGAAGCCTAACTACAGCGAGCTGCAG
Upstream_WT-Bottom strand	/5Phos/TGTAGTTAGGCTTCCGATTGGGGTCGGAGGGCTTCCTGACGAGGAAAGAGCGCGGCATAGTGGTCGAGGCACTGGGGTCGCCGATTCGCGCAGCAGTAGCGCAGAAGAACCACTCGCTAGGCCGTGCCGCGGCGCAATGAATGCAGCAGCGCCGCCAACTCCCTTAAGTACTCCG
Upstream_Bubble-Bottom strand	/5Phos/TGTAGTTAGGCTTCCGATTGGGGTCGGAGGGCTTCCTGACGAGGAAAGAGCGCGGCATAGTGGTCGAGGCACTGGGGTCGCCGATTCGCGCTGTGTCGTACGCTATGCCGTTCTCGCTAGGCCGTGCCGCGGCGCAATGAATGCAGCAGCGCCGCCAACTCCCTTAAGTACTCCG
Downstream-Top strand	/5Phos/GACTCTAATCCAGGTGCGTTGGAGGGGTTCTGGGCTCCAGGAGGTTTGGGGGAGACAGGCGAAGGCTGCGTGGGGGCACCTGAGGGAGGCGGCCTGCCTGAGCCAGGATCGAGTCACAGGATGTTTTGTGGACCATTGCGGGCT
Downstream-Bottom strand	AGCCCGCAATGGTCCACAAAACATCCTGTGACTCGATCCTGGCTCAGGCAGGCCGCCTCCCTCAGGTGCCCCCACGCAGCCTTCGCCTGTCTCCCCCAAACCTCCTGGAGCCCAGAACCCCTCCAACGCACCTGGATTAGAGTCCTGCAGCTCGC
